# Adaptive search space pruning in complex strategic problems

**DOI:** 10.1371/journal.pcbi.1010358

**Published:** 2022-08-10

**Authors:** Ofra Amir, Liron Tyomkin, Yuval Hart

**Affiliations:** 1 Faculty of Industrial Engineering and Management, Technion - Israel Institute of Technology, Haifa, Israel; 2 Department of Psychology, Hebrew University of Jerusalem, Jerusalem, Israel; Harvard University, UNITED STATES

## Abstract

People have limited computational resources, yet they make complex strategic decisions over enormous spaces of possibilities. How do people efficiently search spaces with combinatorially branching paths? Here, we study players’ search strategies for a winning move in a “k-in-a-row” game. We find that players use scoring strategies to prune the search space and augment this pruning by a “shutter” heuristic that focuses the search on the paths emanating from their previous move. This strong pruning has its costs—both computational simulations and behavioral data indicate that the shutter size is correlated with players’ blindness to their opponent’s winning moves. However, simulations of the search while varying the shutter size, complexity levels, noise levels, branching factor, and computational limitations indicate that despite its costs, a narrow shutter strategy is the dominant strategy for most of the parameter space. Finally, we show that in the presence of computational limitations, the shutter heuristic enhances the performance of deep learning networks in these end-game scenarios. Together, our findings suggest a novel adaptive heuristic that benefits search in a vast space of possibilities of a strategic game.

## Author summary

Search problems usually have a common trade-off between accuracy and computational resources; Finding the best solution usually requires an exhaustive search, while limiting computations usually decreases the quality of solutions. Yet, humans provide high-quality solutions for complex problems despite having limited computational resources. How do they do that? Here, we analyze people’s behavior in a strategic game of “k-in-a-row” that has an enormous space of possibilities. We find that people strongly prune the search space by using scoring strategies to evaluate each possibility and augment this pruning with a shutter heuristic that limits their search to the possible winning paths from their last move. Similar to other adaptive heuristics, the shutter heuristic provides a strong reduction in the computations the searcher needs to carry out while maintaining on par accuracy rates. Finally, this adaptive heuristic generalizes to the performance of deep learning networks when playing with limited computational resources.

## Introduction

Making decisions is hard. Making good decisions is even harder. Yet people make strategic decisions almost every day. From marital and career choices to the decision of where to get lunch, many of our decisions are made over a vast space of interdependent possibilities which form a tree of branching decision sequences. What are the cognitive mechanisms that support the search for solutions on such enormous tree-like spaces?

Due to its importance, mapping the cognitive mechanisms that support strategic planning and decision making is an ongoing quest [1–11]. A fundamental approach to the study of strategic planning and decision making is to treat the underlying cognitive mechanism as an information processing system that performs a search in the space of all possible solutions [12]. When searching a branching tree of possibilities there are two layers to the search—First, evaluating the value of the current possibilities which we term the scoring strategy. Second, deciding which possibilities to further explore in order to gain more information. This exploration process can be either random or directed. In the latter case, it usually involves ignoring parts of the space which we term pruning. Thus, any mapping of cognitive search mechanisms should address these two layers of the search.

One line of studies of cognitive search processes suggested that people’s cognitive search is akin to random sampling of the search space [13–16], similar to Markov Chain Monte-Carlo (MCMC) sampling processes [17]. This framework proved highly useful in the description of many cognitive processes—learning of rules [13], theories [14], language [15], and intuitive physics [16], to name a few. However, this type of search demands high computational resources and long computation times. It thus begs the question of how these processes may be relevant when the search is done over enormous spaces of thought under time and computational constraints [18–21].

Recent works on planning and decision making have begun to tackle the question of how people handle extremely large search spaces. These works propose pruning mechanisms that limit the computations involved in the search for the correct action. For example, Huys and colleagues show that people prune the branches of the tree of possibilities that lie after a large loss, even if that behavior results in sub-optimal rewards [5] and that there’s an interplay between mechanisms of fragmentation, stochastic memoization, and losses pruning that trade accuracy and computational costs during the search process [6]. Another line of studies [8, 22, 23] suggests arbitrating mechanisms which toggle between goal-directed and habitual evaluations based on current information. A goal-directed, model-based planning produces a more accurate decision but is time consuming since it searches deep into the tree of future possibilities. Habitual evaluations, on the other hand, are considered model-free and take into consideration only the estimated value of the next action, without searching the tree of future possibilities (and thus saving computations). These works suggest that people arbitrate between goal-directed and habitual estimations as well as decide which directions of the search tree to further expand (plan-until-habit) by comparing the benefit of an accurate response and the reduction of uncertainty in estimations with the cost of the loss of reward caused by the longer search time.

A fruitful avenue to study these questions is the analysis of people’s behavior in complex strategic games. Games like chess [24, 25], Go [26], and Tic-Tac-Toe [27, 28] provide a rich tree-like search space. Each game state maps to a node in the search tree, with the different possible actions leading to alternative future states, which in turn also branch out to different sequences depending on the chosen actions. Importantly, these search trees have an exponential number of branching possibilities of different values, and thus typically cannot be fully explored. Previous studies explored chess players’ quality of moves, recall of game states, and behavior under time pressure to identify key differences between experts and novices [24, 29, 30]. Of special note are recent works [27, 28] that used a “4-in-a-row” game as a platform to study players’ sequential decision making throughout the game, and modeled players’ search as a tree-search process. The search is based on an iterative “best-first” search algorithm that scans possible moves from a given state, and prunes all states that are below the best move value minus a threshold. The scoring strategy for each board state is estimated by a weighted linear combination of benefits from creating sequences of connected squares (and also not-connected 2 squares that might create a winning path) and the value of each square occupied by the player’s pieces. This gain is then subtracted by the possible loss due to similar sequences created by the opponent. Importantly, in all these experimental paradigms, the features of the search are inferred from the actual moves participants decided to play. Their search trajectories, however, are not directly observed and thus key parameters of the search remain mostly unknown.

Our study focuses on the computations of end-game scenarios in the strategic game, “k-in-a-row”. To infer the features of people’s search in this large search space, we map the specific trajectories of people’s search in finer details, going beyond the observation of only the end decisions of their search (i.e. the decision on the next move). We do that by asking participants to find a winning move in different configurations of “k-in-a-row” boards using a ‘sandbox’ board where they can try different moves before committing to the chosen move. We find that the number of nodes participants explore in their search (i.e. their search size, which is measured by the number of moves they tried on the sandbox board) is limited and their accuracy rates fall much slower than the massive increase in the algorithmic complexity of the boards. In their search, participants combine two layers of pruning—First, participants use scoring strategies to evaluate the different moves. Second, participants augment their pruning by focusing the search on potential sequences for winning enabled by their last move. This “shutter” heuristic leads to an inherent blindness to the opponent’s winning moves, and thus bears costs. However, we show that the shutter heuristic outperforms other strategies in the context of “k-in-a-row” end-game scenarios. We scan different shutter size values with a broad range of computational conditions (e.g., complexity levels, noise levels, branching factor, and computational limitations) which demonstrate the dominance of the shutter pruning heuristic for the majority of these conditions. Lastly, we find that the shutter heuristic greatly enhances the winning rate of deep learning neural networks in the game when their computational resources are limited.

## Results

### The experimental task

**Fig 1:**
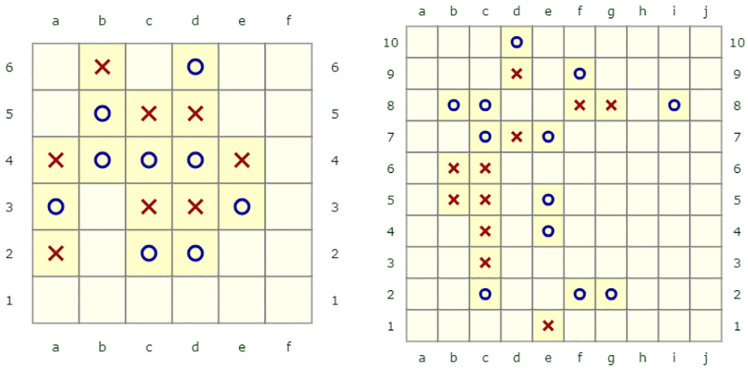
Examples of board configurations. Left, board I (6x6 squares) full condition, ‘X’ wins within 4 moves (correct solutions are ‘f3’ or ‘f5’). Right, board IV (10x10 squares) full condition, ‘X’ wins within 5 moves (correct solution is ‘e8’).

Each participant (Amazon mechanical Turk, N = 915, see Methods) saw one of ten different board configurations. The ten boards are composed of five boards with varying difficulty (two 6x6 boards and three 10x10 boards, denoted boards I–V, see Fig 1, Methods and S1 Fig) and five additional versions of the same boards where we added the next best move for both the ‘X’ and the ‘O’ players. This resulted in truncated versions of the boards with one move toward the solution already revealed. Participants had to find the move that will force a win for the ‘X’ player within 3 (6x6 truncated boards), 4 (6x6 full boards or 10x10 truncated boards), or 5 (10x10 full boards) moves. Participants interacted with a ‘sandbox’ board where they saw the initial board configuration and searched for the winning move by simulating ‘X’ and ‘O’ moves. We recorded all participants’ moves, timings, and whether their answers were correct and validated (see S1 Text and S2 Fig for the analysis of the time intervals between moves). We defined the number of nodes explored by participants as the size of their search (that is, the number of clicks they made on the sandbox board during their session) and for each board configuration, we defined board complexity as the number of nodes an optimal tree search algorithm (alpha-beta pruning search [31], see Methods) needs to explore to find the correct solution.

### Participants’ success rate declines much slower than the increase in board’s algorithmic complexity

**Fig 2:**
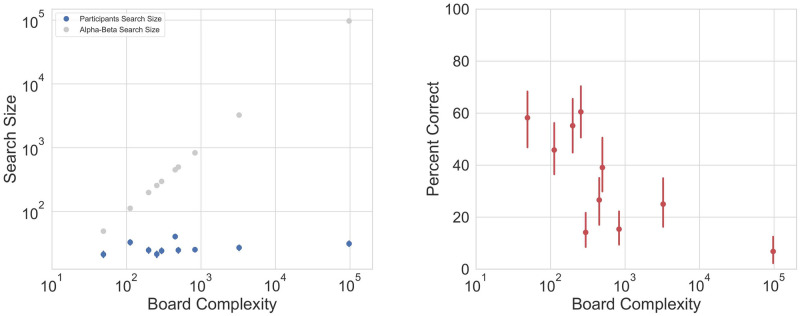
Participants search size does not depend on the board’s complexity. **A)** Participants’ search size (blue) is small and fixed compared with the search size of alpha-beta pruning (gray). **B)** The percent of participants who solved correctly each of the board configurations, by board complexity. As board complexity increases, participants’ success rate decreases, yet at a slower rate compared to the board’s complexity increase. Error bars depict 95% confidence intervals.

We found that while board complexity varied across ∼2000 folds (49 to 97098 moves), participants’ search size varied across ∼2 folds (21–40 moves) and did not show a clear relation with board complexity (Spearman correlation, r = 0.08, 95% CI = [0.01,0.14], *p* = 0.018, see Fig 2A, blue dots vs. gray dots). Similarly, there was no correlation between board complexity and search time (Spearman correlation, r = 0.02, 95% CI = [-0.05,0.08], *p* = 0.56, see S3 Fig which also shows that the limited search is not due to time limitations). Consequently, the rate of successful solutions decreased with the complexity of the board (Spearman correlation, r = -0.31, 95% CI = [-0.37, -0.25], *p* < 0.001), yet success rate decreased by 2–3 folds over an increase in complexity of 3 orders of magnitude (Fig 2B).

We note that since participants were recruited from Amazon mTurk, there might be an incentive to finish tasks quickly and reduce the number of moves in the task. However, the slower decline of the success rate compared to the large increase in algorithmic board complexity may suggest that participants prune the search space to overcome the increased complexity of the problem. What then are the computational pruning mechanisms that facilitate finding the correct solution despite the limited search size?

### Participants use scoring strategies in their search

We hypothesized that participants’ limited tree search can be partially explained by the use of scoring strategies that reflect the deep structure of the game. Each scoring strategy serves to evaluate the quality of moves, thus enabling participants to explore more promising nodes in the tree and to avoid the exploration of low scoring nodes. We evaluated five different scoring strategies (see Methods and S1 Text, S4 Fig, and Table A in S1 Text for the evaluation of these scoring strategies):

1. *Density*: A square’s score is the number of its neighboring squares marked by ‘X’.
2. *Linear*: A square’s score is the sum of scores of its potential winning paths, where each path score is the number of squares with ‘X’ in it. Thus, a square on a path of 2 ‘X’s and another path of 3 ‘X’s will have a score of 5.
3. *Non-linear*: Same as *Linear* but scores increase non-linearly with the number of ‘X’s in the path: $=\frac{1}{n-n_{i}}$ (where *n* is the number of ‘X’s needed to win, and *n*_*i*_ is the number of ‘X’ in path *i*). In a ‘5 in a row’ board, the same square as in the *Linear* example will have a score of $\frac{1}{5-2}+\frac{1}{5-3}=5/6$.
4. *Interaction*: Same as the *Non-linear* scoring, augmented by an interaction term between the shared paths. The added score is $\frac{n_{i}\cdot n_{j}}{{\left(n-1\right)}^{2}-n_{i}\cdot n_{j}}$. For example, the same square as in the *Non-linear* example, will receive an additional score of $\frac{2\cdot 3}{{\left(5-1\right)}^{2}-\left(2\cdot 3\right)}=0.6$.
5. *Forcing*: This scoring is similar to *interaction*, but includes an additional component: if placing an ‘X’ in the square results in an immediate threat (a path that now contains *n* − 1 ‘X’ markers with a potential to win in the next move), the score of the square is augmented by a large constant (10 points) since by forcing ‘O’ to a particular move, ‘X’ essentially blocked all other paths for ‘O’ and controls the game flow.

To assess the total score of each square for e.g. the ‘X’ player, we consider the possible benefits for ‘X’ from gaining that square and add to it the incurred costs for the ‘O’ player from missing that square (see Methods, Eq 2). Thus, each square receives scores from all the gained paths with ‘X’ marks going through this square and all the scores from the prevented paths with ‘O’ marks going through this square. We further note that because the algorithm compares squares’ scores within a scoring strategy, it is the relative scores of different squares that are important rather than their absolute values in each scoring strategy (see also Table A S1 Text).

**Fig 3:**
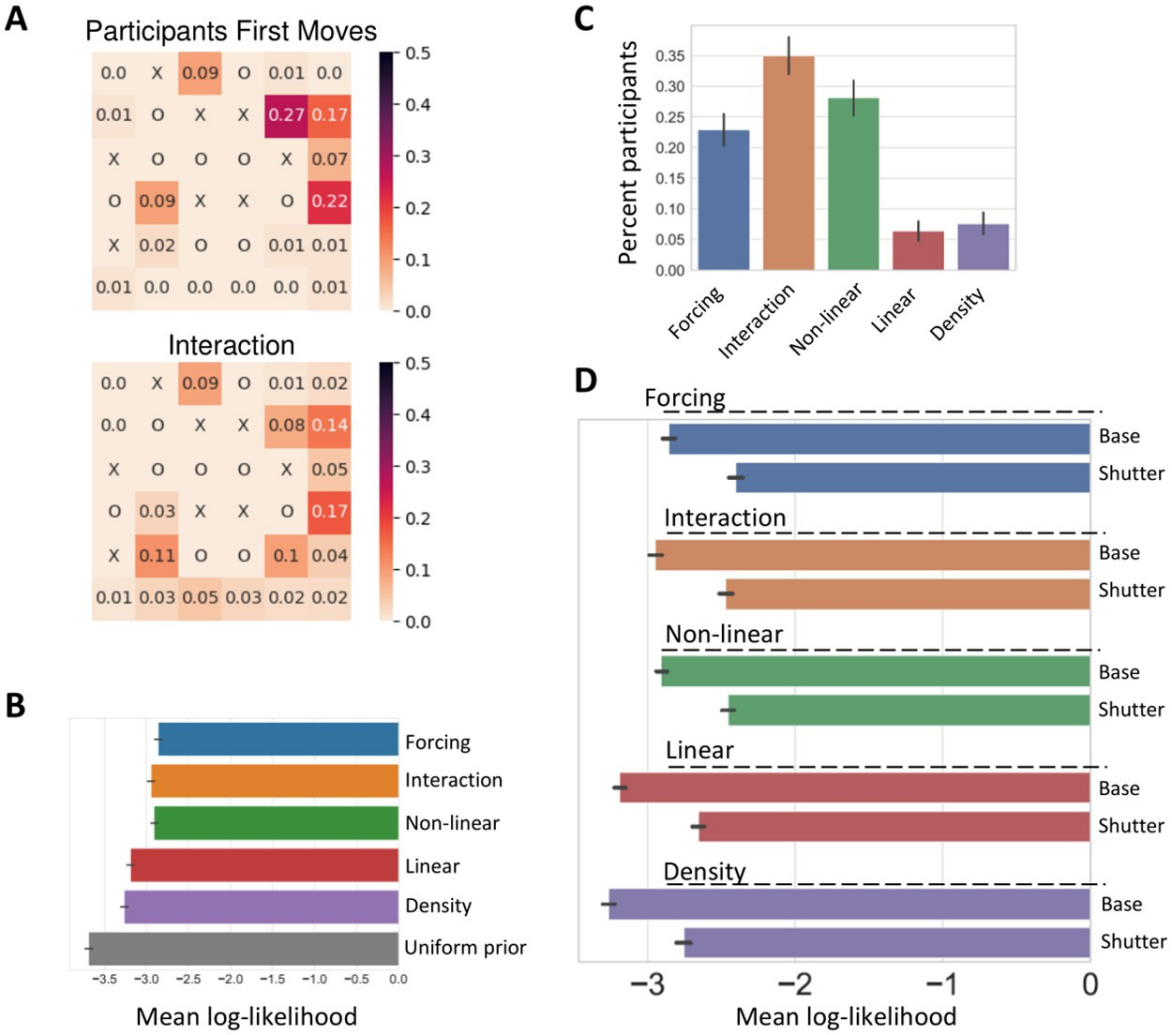
Participants’ moves indicate the use of scoring strategies. **A)** The distributions of first moves predicted by the “Interaction” scoring strategy and the distributions of participants’ actual first moves on this board. **B)** Log-likelihoods of scoring strategies’ predictions of participants’ moves. **C)** Percent of participants fitted to each scoring strategy. **D)** Log-likelihoods of scoring strategies’ predictions of participants’ moves. There are two configurations for each scoring strategy: using the scoring strategy as is (“base”) and adding the best fitted shutter value per each participant (see Methods). For all scoring strategies, adding the shutter significantly improved the log-likelihood and AIC scores (in all cases *p* < 10^−5^ using a likelihood ratio test). All error bars are 95% confidence intervals.

The experimental paradigm allows us to further examine the search pattern itself, meaning the trajectories of moves on the board that the participants chose to explore. We next calculated the probability distribution of participants’ moves and compared it with the distribution induced from the different scoring strategies (see Fig 3A for an example of the distribution of participants’ first moves compared to the prediction of the “Interaction” scoring strategy, and S6 Fig for the distributions in all board configurations).

The distribution of participants’ moves reflects participants’ internal values of the squares on the board. To calculate the similarity between the behavioral data and each scoring strategy’s predictions we computed the log-likelihood of the move probabilities based on the different scoring strategies, with a lapse rate parameter fitted to each participant (see Methods), and then computed the Akaike information criterion (AIC) to choose the best fitted model to participants′ search trajectories (see Methods for more details).

We find that the predictions of the more sophisticated scoring strategies: “Forcing”, “Interaction” and “Non-linear” are closer to participants’ choices than the simpler scoring strategies (Mean log-likelihood and 95% CI (closer to zero is better), “Density”: mean = -3.27 (AIC = 5978), 95% CI = [-3.31, -3.22], “Linear”: mean = -3.19 (AIC = 5837), 95% CI = [-3.23, -3.15], “Non-linear”: mean = -2.91 (AIC = 5324), 95% CI = [-2.95, -2.87], “Interaction”: mean = -2.95 (AIC = 5395), 95% CI = [-2.99, -2.91], “Forcing”: mean = -2.86 (AIC = 5236), 95% CI = [-2.9, -2.81]; see Fig 3B, see S5 Fig for a comparison of log-likelihood scores using Monte-Carlo Tree Search, and see S9 Fig for a sensitivity analysis of the “Forcing” strategy free parameter).

To examine the distribution of scoring strategies among participants, we also calculated the percent of participants that employ each strategy. For each participant we simulate all models based on the different scoring strategies with a fitted lapse rate parameter to account for noisy choices (see Methods). We match for each participant the scoring strategy with the best AIC score (see Methods). We find that for more than half of the participants, the “Interaction” and “Forcing” scoring strategies provided a better fit (58% of the population). A minority of participants were using the simpler scoring strategies, termed “Density” and “Linear” (Percent of participants, Density: mean = 8%, 95% CI = [6%, 9%], Linear: mean = 6%, 95% CI = [5%, 8%], Non-Linear: mean = 28%, 95% CI = [25%, 31%], Interaction: mean = 35%, 95% CI = [32%, 38%], Forcing: mean = 23%, 95% CI = [20%, 25%], see Fig 3C and additional analyses in S1 Text and S7 Fig). Since the “Interaction” scoring strategy provides better fits for most participants (Fig 3C) and has one less parameter in its model compared to the “Forcing” scoring strategy, next sections use the “Interaction” strategy as the base scoring strategy model (unless stated otherwise).

Both participants’ search size and their entire search trajectories indicate that participants search using scoring strategies. These scoring strategies combine non-linear scores of each path and interactions between different paths to allow participants to vigorously prune the search space. Next, we tested whether players augmented their search with additional pruning mechanisms.

### Participants’ previous moves influence their current search choices

Another possible mechanism for pruning the search space is using the memory of previous moves (and previous calculations), instead of deciding anew for each game state as the game evolves. To test whether participants’ search patterns exhibit memory (i.e., an influence of previous moves on future moves), we compared participants’ distribution of moves from a given board configuration in two different conditions—one distribution is the distribution of moves in the truncated board (where participants see the board after one optimal move for both the ‘X’ and ‘O’ players) and the second distribution is participants’ distribution of moves on the full board after participants chose the same optimal first moves for ‘X’ and ‘O’ (as in the truncated version). If players are not influenced by their previous choices, the distribution of moves in the full and truncated boards should be similar.

**Fig 4:**
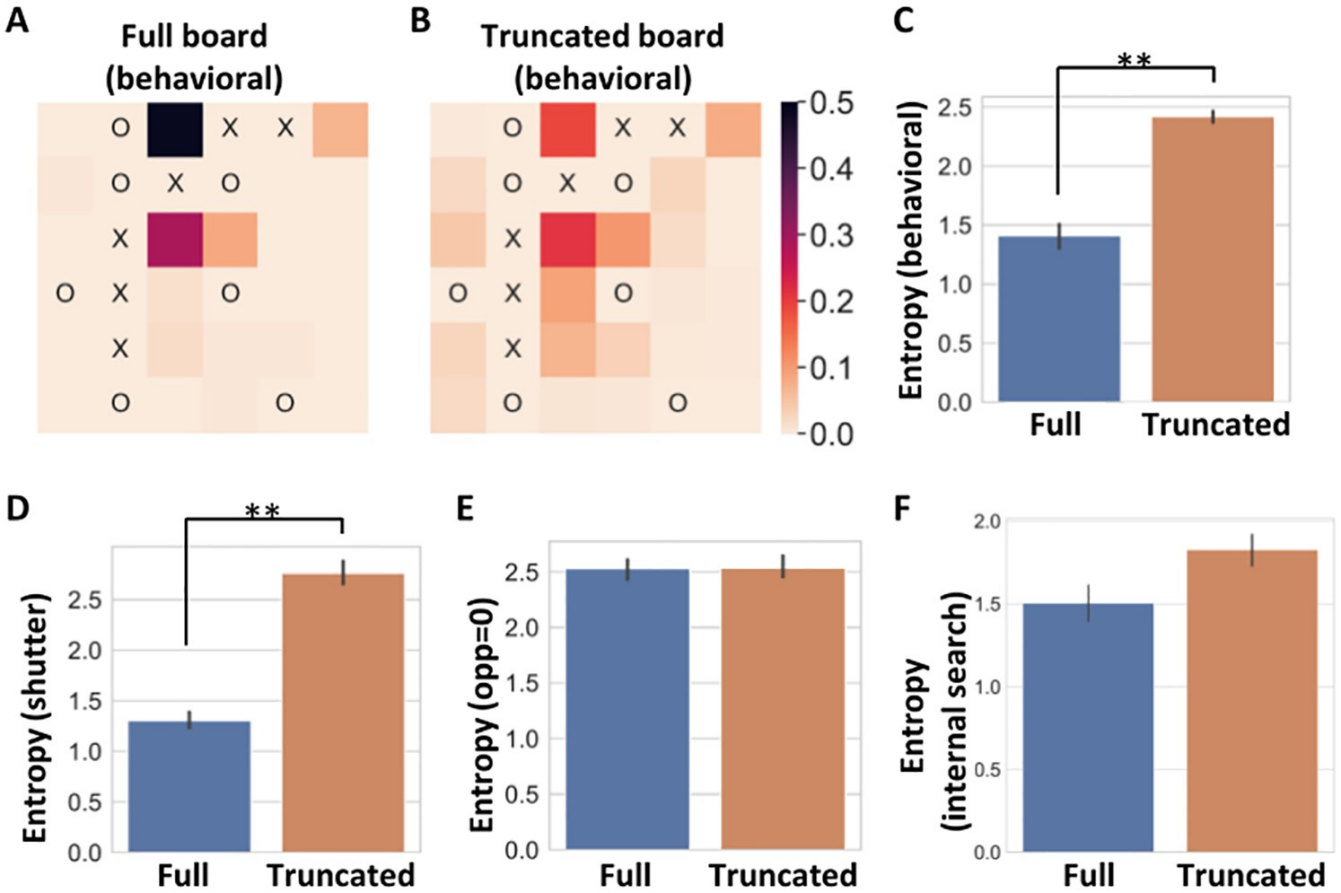
Participants’ search is influenced by their previous choices and is not explained by an internal search model or by reduced attention to the opponent. **A)** Distribution of participants’ moves on the full version of board configuration II. **B)** Distribution of participants’ moves on the truncated version of board configuration II. **C)** Mean entropy of the distribution of participants’ first moves in the truncated boards and the distribution of moves in the equivalent board state in the full boards. **D)** Mean entropy of the distribution of participants’ first moves in the truncated boards and the distribution of moves in the equivalent board state in the full boards as predicted by the “Interaction” scoring strategies with an added shutter heuristic (shutter = 0). **, *p* < 0.01. **E)** Mean entropy of the distribution of participants’ first moves in the truncated boards and the distribution of moves in the equivalent board state in the full boards as predicted by the “Interaction” scoring strategy when ignoring opponent’s winning paths, thus focusing only on one’s own pieces. **F)** Mean entropy of the distribution of participants’ first moves in the truncated boards and the distribution of moves in the equivalent board state in the full boards as predicted by an internal search model, using alpha-beta pruning search with branching factor *k* = 7 and limited depth *d* = 1. All error bars are 95% confidence intervals.

We find that the distribution of moves in the two conditions was highly different (Fig 4A and 4B). When comparing the entropy of participants’ moves in the full and truncated conditions of the same board configuration (see Methods), we find higher entropy for participants’ moves in the truncated board than in the full board (full: mean entropy = 1.41, 95% CI = [1.30, 1.50], truncated: mean entropy = 2.42, 95% CI = [2.37, 2.46], around 40% entropy reduction, Mann-Whitney test U = 0, *p* = 0.006, Rank biserial correlation = 1, Fig 4C). This finding suggests that participants’ choice of the next move depends on the moves made before it. In particular, the lower entropy for moves in the full board suggests that as participants explore a specific search trajectory further, they narrow down the moves they consider (see S10, S11, S12 and S13 Figs for additional analyses and comparisons with the dynamics of a Monte Carlo tree search algorithm).

We note that comparing the entropy of participants from the full board to the entropy of participants from the truncated board might introduce a selection bias: Participants from the full board are required to play two optimal moves before reaching the same board configuration as that of the truncated board. Thus, while in the truncated board all types of participants are represented, in the full board, only less noisy participants (i.e. those that reached the optimal position) are represented. One might then suspect that the entropy difference is caused by a selection bias. To test this, we chose for each participant her best fitted model and simulated either three moves on the full board or one move on the truncated board. In the full board, we keep only the simulated moves that reached the truncated board’s configuration (i.e. did two optimal moves before the third move). We then calculated the entropy over the moves’ distribution for each board and averaged all entropy values across all boards. We find that the selection bias shows a small entropy reduction (around 7%) that cannot explain the entropy difference in the behavioral data (full board entropy: mean = 3.23, 95% CI = [3.23, 3.23], truncated board entropy: mean = 3.47, 95% CI = [3.47, 3.47], see also section 10, S1 Text and S11 Fig). As another test, we compared the entropies of only the participants which solved correctly the board configurations in both full and truncated boards. This comparison focuses on participants on the two boards that found the optimal path and so are probably less noisy. This analysis produced similar results as the entire behavioral data (see S11 Fig). These two analyses suggest that the entropy difference in the behavioral data does not stem from a selection bias. Another option is that participants use different scoring strategies due to the different complexities of the two board configurations, however, we did not find a difference in the distribution of the fitted scoring strategies between the two conditions (see S8 Fig).

### A path shutter prunes the search space and predicts players’ blindness to winning ‘O’ moves

The influence of previous moves on current choices suggests that participants use additional pruning mechanisms beyond the use of scoring strategies. We hypothesized that this pruning is done via a mechanism of a path *shutter* heuristic which focuses the search to potential winning sequences stemming from the last move in the search tree.

**Fig 5:**
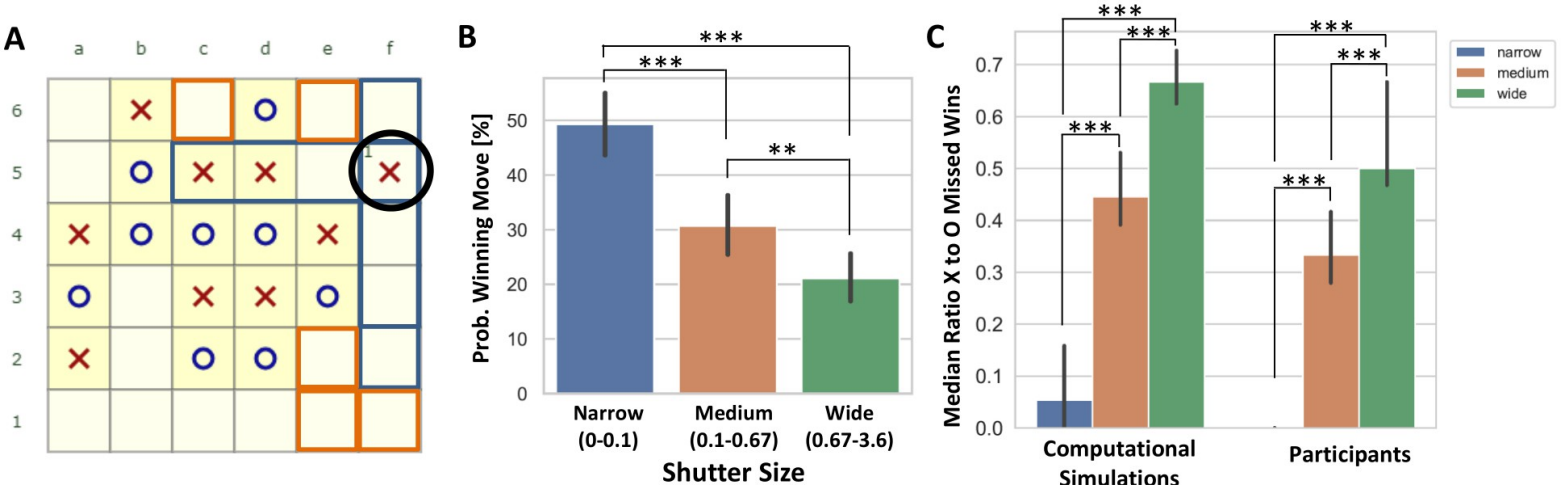
Participants who exhibited a narrower shutter were more likely to find the winning move, but were also more likely to miss winning moves for the ‘O’ player. **A)** Illustration of the shutter heuristic: assuming the player’s last move was f5 (shown in a black circle), there are three potential paths to win induced by this move, f6–f3, f5–f2 and c5–f5 (their squares marked in blue). Squares on these paths are considered at distance 0 from the last move. Squares adjacent to these squares (Manhattan distance = 1) are considered at distance 1 from the last move (marked in orange). **B)** The probability to find the winning move of participants with different shutter sizes. All differences were statistically significant: **, *p* < 0.005 ***, *p* < 0.001. **C)** The proportion between the probability for missed ‘X’ winning moves and the probability of missed ‘O’ winning moves in the computational simulations (left) and by participants (right). Narrow shutter shown in blue, medium in orange and wide in green. Differences between narrow and wide shutter size and medium and wide shutter size were statistically significant, ***, *p* < 0.001. All error bars are 95% confidence intervals.

We measured shutter size by calculating the distance of a move from the potential winning sequences induced by the previous ‘X’ move, such that moves that are part of a currently possible winning sequence are considered at zero distance, the moves next to them (Manhattan distance = 1) are at distance 1 and so on (see Methods and Fig 5A).

The shutter heuristic can explain the differences in entropy between equivalent full and truncated board states observed in the behavioral data. To that aim, we used the “Interaction” scoring strategy with a shutter heuristic (shutter = 0) to simulate moves on the board and compute their expected entropy. We observed a reduction in entropy in the full boards compared to the truncated boards which is similar to that observed in the behavioral data (full: mean entropy = 1.3, 95% CI = [1.23, 1.39], truncated: mean entropy = 2.76, 95% CI = [2.66, 2.88], around 50% entropy reduction, Fig 4D).

We further find that participants exhibiting a smaller shutter size in their search (hence, more focused) were more successful at solving the given board configuration (Success rates, the number of trials solved correctly out of the total trials, narrow shutter: mean = 0.49, 95% CI = [0.44, 0.55]; medium shutter: mean = 0.31, 95% CI = [0.25, 0.36]; wide shutter: mean = 0.21, 95% CI = [0.17, 0.26]; Statistical significance, narrow vs. wide: Mann-Whitney test U = 31114, *p* < 0.001, Rank biserial correlation = 0.28; narrow vs. medium: Mann-Whitney test U = 37356, *p* < 0.001, Rank biserial correlation = 0.19; medium vs. wide: Mann-Whitney test U = 39967, *p* = 0.004, Rank biserial correlation = 0.1; see Fig 5B). We note that the success rates are fairly low despite the solution being within a few moves. Yet, since the searched space is combinatorial in nature and the branching factor is large, the search space is large as well, making the problem difficult (as is also indicated by the boards’ algorithmic complexity).

However, the shutter heuristic also bears costs—If participants are using a shutter that focuses on moves on potential ‘X’ paths to win, they should miss more ‘O’ winning moves than ‘X’ winning moves (where misses of winning moves are events where a participant could make a winning move, but made another move instead). To infer the effects of a shutter on the probability to miss either ‘O’ or ‘X’ winning moves, we simulated the search while varying the shutter size—As the shutter narrows, fewer squares outside the possible winning paths of ‘X’ are considered (see Methods). Indeed, the computational simulations indicate that as the shutter narrows, the likelihood of missing ‘O’ winning moves increases and the likelihood of missing ‘X’ winning moves decreases, resulting in a decreasing ratio of their likelihoods (Simulations, Median ratio of missed ‘X’ wins to missed ‘O’ wins: narrow shutter: median = 0.05, 95% CI = [0, 0.16], medium shutter: median = 0.45, 95% CI = [0.38, 0.53], wide shutter: median = 0.67, 95% CI = [0.62, 0.73], all differences are significant with *p* < 0.001, Fig 5C, see S1 Text for the statistical tests). Thus, the computational simulations predict that the ratio of missed ‘X’ wins to missed ‘O’ wins grows with shutter size. These predictions agree with the behavioral data—As participants’ shutter size increases, the ratio between missed ‘X’ wins to missed ‘O’ wins grows as well (Participants, Median ratio of missed ‘X’ wins to missed ‘O’ wins: narrow shutter: median = 0, 95% CI = [0, 0], medium shutter: median = 0.33, 95% CI = [0.27, 0.42], wide shutter: median = 0.5, 95% CI = [0.44, 0.67], all differences are significant with *p* < 0.001, Fig 5B, see S1 Text for the statistical tests). This increase in the ratio of missed wins of participants as shutter size increases is driven by both a significant increase in missed ‘X’ wins and a significant decrease in missed ‘O’ wins (see S15 Fig).

We note that the problem of finding a winning move is different from a general search problem since participants know that there is a sequence of moves that necessarily wins. This knowledge may guide participants to adhere to the optimal path to win, and this might show as a shutter heuristic. Another possibility is that shutter moves have intrinsically higher values and thus are chosen. However, participants more frequently chose moves that adhere to the shutter heuristic even when their previous move was not the optimal one (i.e., not on the winning path, % of moves with shutter 0 when previous move was not optimal = 65%). In addition, as exemplified by their blindness to their opponent’s winning moves, participants chose more frequently to adhere to the shutter heuristic even when their current choice was not the optimal one given their current board configuration (50% of moves with shutter 0 were not optimal, see also section S15 in S1 Text and S16 Fig). Similarly, on board V, using the “Forcing” strategy diverts participants from the correct solution, yet they still use it regardless (68% of participants show maximal likelihood for the “Forcing” strategy).

Finally, we examined the fit of participants’ moves to a model that combines the scoring strategies and the shutter heuristic. We find that adding the shutter heuristic significantly improved the log-likelihood of the predictions made by all scoring strategies (When comparing each base model to a model augmented by a shutter parameter, all models significantly improved (*AIC*_*base*_ − *AIC*_*base*+*shutter*_ > 826, and *p* < 10^−5^ using a likelihood ratio test, see Fig 3D).

### An internal search model and adjusted weighting of opponent moves do not explain the observed effect of previous moves

Another possible explanation for the entropy difference between the full and truncated boards is that people use a limited look ahead strategy such as a short mental internal search on the tree before they mark their chosen move on the ‘sandbox’ board. In such a case, participants can re-use previous calculations in their next internal searches. These previous calculations might prune the search space and thus reduce the entropy of moves’ distribution on the third move. To test this, we implemented two internal search models, one that prunes the space via the alpha-beta pruning algorithm and the other that implements a Monte Carlo Tree Search (MCTS) algorithm. The alpha-beta pruning internal search model implements a depth-first search up to depth *d* and uses the scoring strategy to evaluate the nodes it reaches. The algorithm prunes tree branches that show worse performance compared to the already found alternatives. Based on this search, the next move on the board is chosen. For the next move, only branches emanating from this specific tree branch are considered, thus reducing the possibilities of next moves. For the entropy calculation we considered only the results of the chosen moves in the simulation and not the moves in the entire internal search that the simulated agent did to reach the chosen move. We simulated different realizations of the model’s two parameters: the depth of the internal search (with values between 1–3) and the number of nodes considered at each level of the tree (with values between 5–10).

In Fig 4F we show that this model still does not achieve the entropy reduction shown in the behavioral data. The alpha-beta pruning internal search model predicts a lower entropy in the truncated boards (entropy of the best fitting model, full: mean = 1.51, 95% CI = [1.4, 1.61], truncated: mean = 1.83, 95% CI = [1.73, 1.92], around 17% entropy reduction, Fig 4F). However, the reduction in entropy is smaller than that observed in the behavioral data. In particular, while the entropy value in the full boards is similar to that observed in the behavioral data, the entropy in the truncated boards is lower (1.83 for internal search vs. 2.42 in the behavioral data), possibly because of the added internal search. Furthermore, the log-likelihood of the best fitted alpha-beta pruning model are worse than the log-likelihood of the shutter heuristic (log-likelihood of best fitting alpha-beta internal search model: -3.17, 95% CI = [-3.24, -3.09] compared with a log-likelihood of -2.21, 95% CI = [-2.17, -2.25] for the “Forcing” strategy augmented with a shutter model). We also implemented a different internal search model using Monte Carlo Tree Search (MCTS) with a limited search and with a memory of past computations (see Methods). However, this model also does not predict the reduction in entropy between the full and truncated board configurations, and does not fit the behavioral data well in terms of the log-likelihood (see section S11 in S1 Text and S12 and S13 Figs).

The observed effect of “blindness” to the opponent’s winning moves could alternatively be explained by a mechanism that gives more attention to players’ own pieces (‘X’) than to their opponent’s pieces (‘O’). To test whether this model can better explain the behavioral data, we simulated agents with a differential attention on their own pieces than their opponent’s pieces (ranging from equal attention to both players, i.e., 50% attention to the opponent’s pieces, to no attention to opponent’s pieces, with jumps of 10%, see Methods and Eq 2). First, for each participant we took their best fitted strategy and compared the log-likelihood scores of this scoring strategy with the shutter heuristic or with the differential weighting model. The two models achieve similar log-likelihood results (shutter heuristic log-likelihood:-2.12, differential weighting log-likelihood: -2.15, see section S11 in S1 Text and S17 Fig). We then calculated the entropy of moves in the full and truncated boards according to the differential weighting of attention simulations. We find that there is no entropy reduction, i.e., that the entropy of the full board and the truncated board are not significantly different when not paying attention to the opponent’s pieces (mean entropy full: 2.53, 95% CI = [2.43, 2.6], mean entropy truncated: 2.53, 95% CI = [2.46, 2.64]; Mann-Whitney test U = 124695, *p* = 0.47, Rank biserial correlation = 0.002, Fig 4E). Lastly, we note that augmenting the shutter model with a decreased attention to opponent’s pieces improved the log-likelihood fit to the behavioral data, thus indicating that while differential attention cannot fully account for the behavior of participants in the task, it might play a role in the players’ search mechanism (see S17 Fig).

### Path shutter pruning is dominant for most search parameters and board configurations

The use of a path shutter shows both benefits (higher likelihood of solving the problem and smaller search size) and costs (missing potential winning moves of the opponent, leading to a wrong solution). We next examined whether using a shutter heuristic incurs a trade-off between using computational resources and the probability to reach the correct solution. To that aim, we simulated the search strategy using the alpha-beta pruning algorithm with the “Interaction” scoring strategy and varied the size of the shutter (Methods). We ran the algorithm on all ten board configurations (I–V, full and truncated) and varied the search algorithm’s main parameters—The noise levels in the score of each square (noise values ranging from 0–2.5), the branching factor which is the maximal number of expanded squares in each level of the search tree (values ranging from 3–10), and the limit on the maximally allowed search size (values ranging from 30–200). In total, we scanned 1760 different boards and search configurations, each simulation repeated 100 times for a total of 176,000 runs (see Methods).

**Fig 6:**
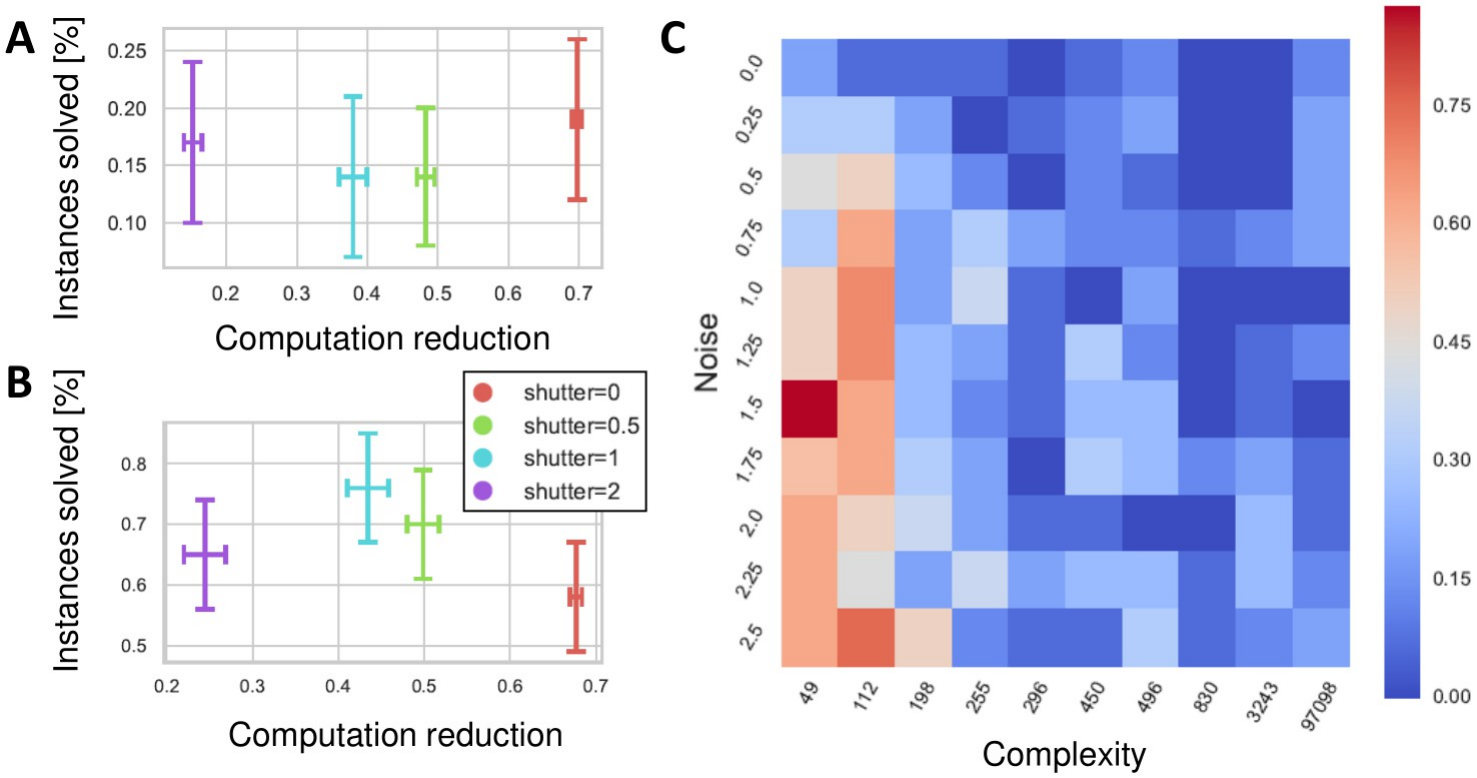
For most of possible search parameters, search with a narrow path shutter is the dominant search strategy. Computation reduction is computed as $1-\frac{computation}{max(computation)}$ **A)** Example of a board configuration where a narrow shutter is the dominant search strategy since it shows similar probabilities for finding the winning move at substantially lower number of computations. Simulation parameters were: complexity [830], noise level [0.5], branching [5], limit number of moves [30]. **B)** Example of a board configuration with a trade-off between computation amount and the probability to find a winning move. For the given simulation parameters, an increase from shutter size 0 to shutter size 1 incurs a significantly higher probability to find a winning move but the number of computations increases as well. Simulation parameters were: complexity [49], noise level [0.5], branching [5], limit number of moves [30]. **C)** The phase space of board complexity vs. noise levels (aggregated over branching factor and search size limitations, see Methods). Each square shows the proportion of configurations in which there was a trade-off between using narrow vs. wider shutter size values: dark blue indicates configurations where the narrow shutter dominates the Pareto front (no trade-off), dark red indicates a trade-off between narrow and wider shutter values (trade-off between computation resources and accuracy). All error bars are 95% confidence intervals.

To assess the optimality of the search, we considered two performance measures: 1) The probability of finding the winning move (accuracy), and 2) The number of score evaluations performed throughout the search (computational resources). For each configuration, we examined whether there is a trade-off between the reduction in computation achieved by the shutter heuristic and the probability of finding the winning move. We consider a configuration to present a trade-off if there was a statistically significant difference (*p* < 0.05, using bootstrap) between the mean values of the two parameters when comparing different shutter values. We find that in most settings, the algorithm with the narrow shutter dominates all other shutter sizes, as it requires substantially lower amounts of computation while not sacrificing the probability to find a winning move compared to larger shutter size values (Fig 6A). Only in a few scenarios, we observe a trade-off between computation size and successful search, where multiple shutter size values lie on the Pareto front (Fig 6B).

A comparison between the states where a narrow shutter is dominant and states where there is a trade-off between shutter sizes indicates that trade-offs are more likely to occur when the complexity of the board is low and noise levels are high (Fig 6C). When squares’ scores are accurately computed based on the scoring strategy (low noise), the path shutter is less likely to hamper performance as the best moves are likely to be selected. For such cases, focusing on the current path reduces computation while maintaining accuracy rate. However, when solving low complexity boards in the presence of higher noise in score evaluations, exploring alternative paths becomes more important and viable, and thus a wider shutter can improve performance. Importantly, for high complexity boards, the probability of finding the winning move is a-priori very low while the probability of following non-beneficial paths is high, thus widening the shutter size substantially increases the computations made and can also lead to deleterious effects by exploring less promising parts of the search space.

### A path shutter heuristic improves the performance of deep learning models with limited computational resources

Lastly, we asked how general are the computational benefits of the shutter heuristic? Would the computational benefits to the cognitive search mechanism of participants generalize also to other computational machines, such as deep neural networks when computational resources limit the amount of possible search? To answer this question, we trained current state-of-the-art models (AlphaZero models, see Methods) that utilize deep learning methods, and examined the effect of using pruning by a path shutter on top of these models while their computational capacity is limited. We used the trained models to play against a Monte-Carlo Tree Search (MCTS) algorithm on the full boards of our experiments, as well as on empty boards (from the beginning of a standard game). We compared the performance of the models when playing as is, to their performance when playing with a path shutter heuristic, averaged across 1000 games. In these games, moves were chosen based on the probabilities predicted by the network, with an additional limited MCTS search, to simulate a scenario with limited computation (0, 25, 50, and 100 simulations, see Methods and S18 Fig).

**Fig 7:**
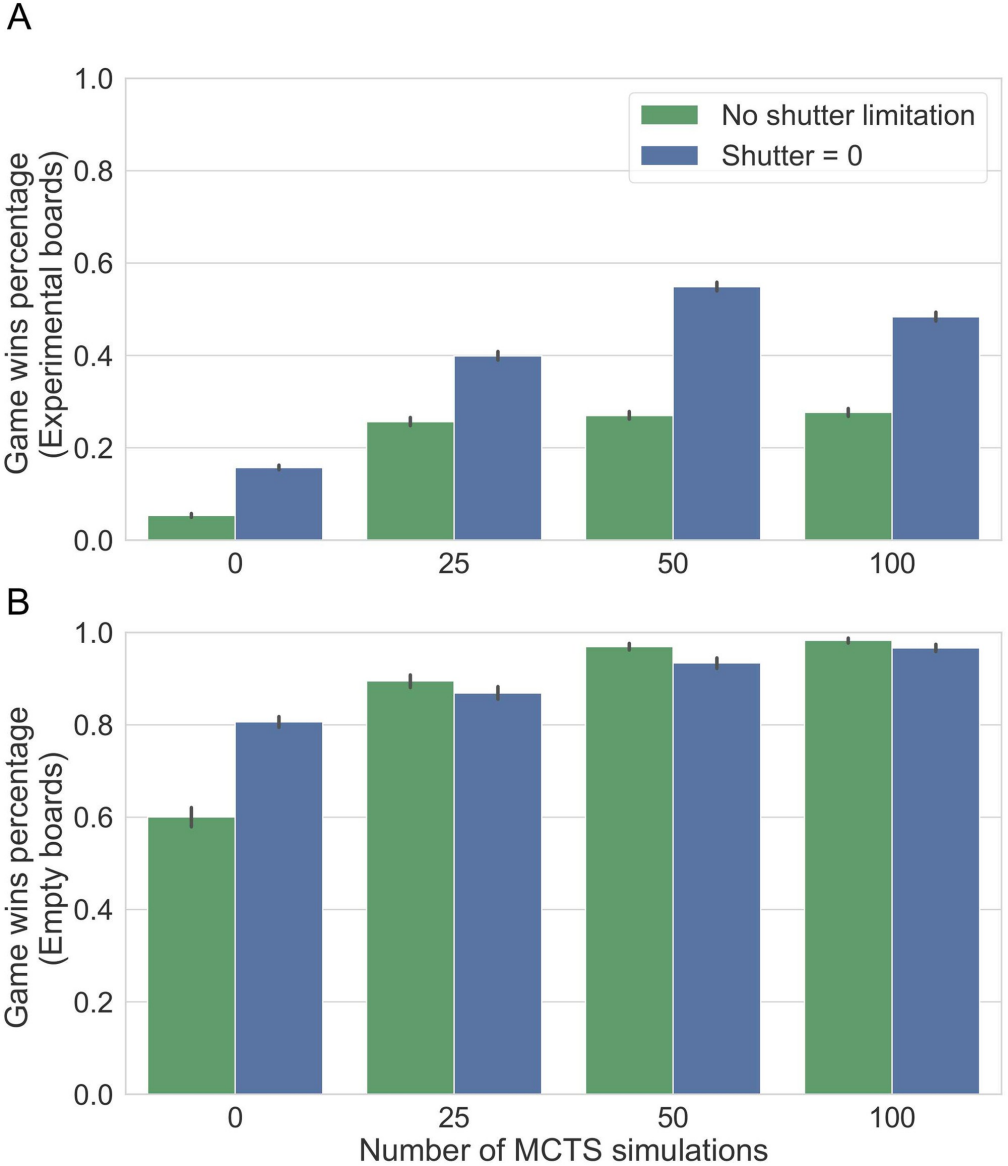
Aggregated results for AlphaZero with different sizes of additional search using MCTS against pure MCTS with 1000 simulations. **A)** Limiting the trained deep learning models with a shutter of size zero (blue bars) significantly improved their performance against an MCTS algorithm on all experimental board configurations (all *p* < 10^−5^). **B)** Adding a shutter heuristic did not improve performance on empty boards (i.e., from the initial state of a game), except when no additional MCTS search was done. All error bars are 95% confidence intervals.

Pruning the search space with the shutter heuristic significantly and substantially improved performance on all experimental board configurations (AlphaZero with no added MCTS: No shutter: 0.05, 95% CI = [0.05, 0.06], With shutter: 0.16, 95% CI = [0.15, 0.16], AlphaZero with 25 MCTS simulations: No shutter: 0.26, 95% CI = [0.25, 0.27], With shutter: 0.4, 95% CI = [0.39, 0.41], AlphaZero with 50 MCTS simulations: No shutter: 0.27, 95% CI = [0.26, 0.28], With shutter: 0.55, 95% CI = [0.54, 0.56], all *p* < 10^−5^, Fig 7A). These findings suggest that the shutter heuristic is adaptive in end-game scenarios of “k-in-a-row” also for deep learning neural networks, otherwise handicapped with limited computations. This benefit of the shutter heuristic was not apparent when playing from the beginning of the game (rather than end games), except for the setting where no additional MCTS search was done on top of the trained network predictions (AlphaZero with no added MCTS: No shutter: 0.6, 95% CI = [0.58, 0.62], With shutter: 0.81, 95% CI = [0.79, 0.82], *p* < 10^−5^, Fig 7B). We note that this may be partially explained by a ceiling effect as all models performed very well (> 90% win percentage) on the empty boards as this is the setting they were trained on. These results demonstrate the ability of the shutter heuristic to enhance the performance of a computational agent with limited computational resources. We note that modern machine learning with no limited computational resources shows performance at a ceiling effect and thus does not gain from the addition of a shutter heuristic.

## Discussion

This study focused on the search strategies people employ in complex scenarios, where the preferred action is embedded in an enormous space of possibilities. We find that participants’ search size did not depend on the complexity of the board. To explain this discrepancy, we suggest two layers of pruning of the search space—One, the use of scoring strategies that captured participants’ search size and their entire search trajectories. Second, a shutter adaptive heuristic that focuses participants’ search on paths emanating from their last move and explains participants’ blindness to winning ‘O’ moves. By scanning a large set of the search algorithm’s parameters and analyzing the Pareto front for each set of parameters, we found that the combination of a scoring strategy with a shutter heuristic is dominant for most of the parameters’ space. Lastly, our findings suggest that the computational benefits of the shutter heuristic generalize from cognitive search of people to deep neural networks with computational limitations.

To infer the features of participants’ search we recorded their search trajectories. Participants marked their moves on a ‘sandbox’ board as they searched for the winning move. The ‘sandbox’ space provides many snapshots of participants’ thinking process and therefore extends the classical “think-aloud” protocols in problem-solving experiments [1, 32, 33], as participants can easily simulate different possibilities directly on the ‘sandbox’ board and do not need to vocalize their thinking process while performing simulations. Thus, our paradigm provides a fine-grained quantitative information on participants’ search trajectories.

The fine-grained mapping of participants’ search suggests that at end-game scenarios participants use scoring strategies as a strong pruning mechanism of the search. This limited search is different from previous suggestions that people use large search sizes on the order of 800–1000 nodes searched per a move when modeling people’s entire game behavior [27, 28]. The use of scoring strategies that embed the structure of the game raises the question of how this structure is learned [34–38]. An interesting avenue for further research is mapping the dynamics that lead to the formation of the non-linear, paths interaction scoring strategies, and to the preference for a narrow shutter. Such shutter heuristics can arise from creating bounded spaces for search [39], from amortization of previous search results [6, 40], or other mechanisms. Our paradigm allows to characterize the scoring strategies and shutter size of each player from their search trajectories, and can thus further promote understanding of how brain activity correlates with individual search strategies.

Our findings reveal a trade-off between the ability to search successfully for a solution and noticing the strategy of an opponent, as expressed by the blindness to winning ‘O’ moves. This effect is mitigated by the shutter size, where a narrow shutter allows for greater success in finding the winning moves for ‘X’ but also more misses of ‘O’ winning moves. Interestingly, for most of the search configurations, this trade-off is not manifested and a small shutter size is the dominant search strategy, strengthening previous claims for the need to assess cognitive function in real-world, uncertain, and noisy environments [18, 41, 42].

The shutter heuristic complements other pruning strategies previously found in human planning and decision making [5, 6, 8, 22, 23, 43, 44], such as fully scanning the tree of possibilities up to a certain depth [43], pruning tree branches after large losses [5], arresting search if the estimated benefit of an accurate response does not outweigh the cost of delaying the decision [8, 22, 23], or choosing which experienced states to replay by their contribution to inferring the policy for maximal expected return [44]. The Pareto analysis suggests that in the specific case of end-game scenarios, the adaptive shutter heuristic is not influenced by the speed-accuracy trade-off (and see also [45–54]).

The Pareto front of the shutter heuristic suggests that the shutter acts as an adaptive heuristic—providing on par accuracy rates with much reduced computations compared with a global scan of the search space. As such, it is part of a growing literature that points to the benefits of stepwise adaptive heuristics (e.g., split-half [11], focus on highest probability [9], undergeneralization [55], likelihood difference [56], positive testing [10], and submodularity [57]) which provide similar performance to global optimal policies at reduced computational costs. Such reduction in computational costs makes these heuristics favorable, as people tend to avoid extra cognitive costs [58–60]. We note that similar to other adaptive heuristics, participants in the task choose to use the shutter heuristic even when it is suboptimal in its performance and there are better choices at hand.

While the experimental design records the search trajectories participants try on the board, it does not record their entire search trajectory on the tree which could cover more of the search space. Still, key features of their search—the independence of search size on the board’s complexity, the influence of previous moves on the search, and the blindness to ‘O’ moves—remain the same and indicate a strong and biased pruning process of the search space. This study focused on end-game scenarios which have the advantage of setting a complex yet tractable search challenge. However, the task instructions may direct participants to use the “Forcing” strategy since they know they can win within a limited number of moves. Future studies should test whether the tendency toward the “Forcing” strategy remains when the instructions are less suggestive in nature. Moreover, the end-game configurations provide rich structures that benefit from the use of scoring strategies and participants are aware that a viable solution exists. However, in the beginning of the game, such scoring strategies and assumptions might be redundant and costly, which might explain the difference in the estimated search sizes between our findings and previous work that estimated entire game behavior [27, 28]. We note that while the scoring strategies players use at the end-game boards are relevant throughout the entire game, the shutter heuristic is relevant only when winning paths become plausible. As such, in the beginning of the game the shutter heuristic is less useful. Since our findings suggest that players do rely on this heuristic at end-game scenarios, an open question is what are the conditions upon which players decide to adhere to the shutter heuristic. It is therefore intriguing to characterize the changes in the use of the scoring strategies and the size of the shutter from the game’s beginning to its end.

Our study utilizes the “k-in-a-row” game which has a strong correlation between the “planning” space (the tree of possible future moves) and the spatial space (the pieces’ location on the board). In principle, the shutter heuristic could be applied directly at the planning space, focusing on the current move and its induced paths to the goal while ignoring all others. As such, it may extend to many other complex strategic planning scenarios. Other strategic games, such as checkers, chess and Go, provide an elaborate tree-like search space with a decreased connection between the tree of possibilities and the spatial location of the pieces on the board. An interesting direction to test the generality of the shutter heuristic is to study end-game scenarios in these strategic games.

The human ability to perform successful search in enormous spaces of possibilities is one of the marvels of human cognition [18–20, 39, 61–64]. Studying in great detail the ways by which humans search vast spaces of decisions, hypotheses, and ideas, can point to specific cognitive computational mechanisms with their benefits and costs. Our findings suggest a novel adaptive heuristic that humans and machines alike seem to benefit from in their search of an immense space of possibilities in a strategic game.

## Methods

### Ethics statement

The study protocol was approved by the Institutional Review Board at the Technion. Written consent to participate in the study was obtained for all participants.

### Participants

We recruited participants through Amazon mechanical Turk. We restricted participation to workers from the US with a high reputation. We used a between-subject design where each participant was only allowed to complete one task to avoid learning effects. Participants received a base payment of $1.5 and earned an additional bonus of $1 if they correctly solved the problem. We removed 107 participants from the study who performed fewer than 4 moves in their search for a solution, yielding 915 valid participants (*N* = 915, age: mean = 35.5, SD = 10, 405 females). 18% of the excluded participants solved the task correctly.

### Task

We asked participants to find the winning move that will force a win within a given number of moves on a “k-in-a-row” board configuration. The “k-in-a-row” game provides an opportunity to map decision making processes in a rather heterogeneous and broad population of participants. We constructed five board configurations which differed in their difficulty, as reflected by the number of moves a naive search algorithm takes to find the solution (Fig 2). Two of the configurations were ‘4 in a row’, 6x6 boards, and three configurations were ‘5 in a row’, 10x10 boards (see Fig 1). These board configurations have a ground-truth solution, but finding this solution is challenging due to the size of the search space. The boards were generated semi-automatically—we had a search algorithm play against itself, and added noise to the algorithm to allow for different outcomes. We then searched for board configurations from which one player can force a win within 4 or 5 moves. Next, we manually chose two 6X6 configurations, based on which we also created two 10X10 configurations (by extending the boards in a way that maintains the same basic configuration but extends it to a 5-in-a-row game). We additionally selected another 10X10 configuration (board V) from the algorithm’s games. This board was chosen to test participants’ search dynamics when the most obvious “Forcing” first move does not actually produce a win within the required number of moves. For each of the five board configurations, we generated an additional task in which we showed participants a version of that board with an additional optimal move for ‘X’ and ‘O’. In these *truncated* configurations, participants had to indicate the winning move within 3 or 4 moves (compared with 4 or 5 moves in the *full* version respectively). The truncated boards allow us to compare participants’ choices in identical board states, with or without a previous move being made and thus to infer whether the search depends on previous choices or not. All board configurations are shown in S1 Fig.

Participants were instructed to find the winning move for ‘X’, e.g., “In this position X can force a win in 4 turns. This means that X can win the game by placing 4 more marks, even when O plays to win as well.” (see S1 Text for the complete experimental procedure). They were provided with a ‘sandbox’ GUI for searching for the winning move. Using the GUI, they could simulate moves for both themselves (‘X’) and their ‘opponent’ (‘O’) by clicking on squares on the board. They could also reset the board to its starting position or undo their last move using the ‘reset’ and ‘undo’ buttons. All participants’ actions (clicks, undo and reset) were recorded. During their entire game, participants do not receive any feedback on their search or submitted solution. To verify that participants did not guess the correct solution, after submitting their answers, we asked them to play their solution against the computer.

The task can be accessed at the following link: http://ec2-54-149-86-189.us-west-2.compute.amazonaws.com/webTTT/public_html/tictactoe/tictactoe.html?board=1f. The code for the web experiment can be found here: https://github.com/OptimalSearchSpacePruning/k-in-a-row-web-experiment.

### Measuring board complexity with the alpha-beta pruning algorithm

The “k-in-a-row” strategic problems used in our experiments belong to the P-SPACE complete computational complexity class [65]. To quantify the complexity of solving each board, we implemented an optimal tree-search algorithm for two-players games called alpha-beta pruning [31]. In general, tree search algorithms utilize two main mechanisms: (1) evaluating the value of a node, and (2) deciding which nodes to explore further. The first mechanism typically utilizes a “scoring strategy” that quantifies the value or “goodness” of that state. For example, in k-in-a-row, the scoring strategy can consider the number of potential winning paths currently occupied by a player. The decision of which nodes to explore further is typically dictated by the scoring strategy, but can also consider other pruning mechanisms such as exploring the tree only up to a certain depth, and how many different paths to explore. Alpha-beta pruning traverses the search tree in a depth-first manner assuming optimal play from both players, and prunes branches of the tree that could not outperform the best solution obtained so far. We limited the algorithm’s depth of search to the predefined solution depth (3, 4 or 5 ‘X’ moves to win, according to the board configuration). We used a scoring strategy for quantifying the value of terminal board configurations, as well as to rank the possible nodes such that more promising nodes will be expanded first (similar to best-first search methods). As such, alpha-beta pruning adds to the random tree search methods knowledge about the structure of the board, reflected by the scoring strategy. We used the “Interaction” scoring strategy to evaluate the search size for each board.

### Computing log-likelihood of scoring strategies’ predictions

To assess the fit of the scoring strategies to participants’ behavior, we computed the log-likelihood of participants’ moves based on the predictions of the scoring strategies. For each scoring strategy, we computed a probability distribution over moves for each game state. Since many moves might receive a score of zero, we fitted a lapse rate parameter (*ϵ*, values between 0–0.05 in jumps of 0.01) for each participant. That is:

$$\begin{array}{c} L\left(pos_{j}|board,h\right)=log(\left(1-\epsilon \right)\cdot prob_{h}\left(pos_{j},board\right)+\frac{\epsilon }{\#\text{legal}\;\text{moves}}) \end{array}$$ (1)

Where *prob*_*h*_(*pos*_*j*_ , *board*) denotes the probability of placing an ‘X’ or an ‘O’ on a particular square position *pos*_*j*_ using a scoring strategy *h* in a particular board state. This probability is computed by dividing each score with the total scores over all squares on the board (moves with negative scores are assumed to have zero probability). We then computed the mean likelihood of the moves performed by each participant. We note that “undo” or “reset” actions are not explicitly modeled, they simply change the state of the board which is then reflected in consequent moves.

### Fitting scoring strategies to participants

We calculated the percent of participants in the population that employed each scoring strategy. For each participant we fit for each scoring strategy the best shutter value (ranging between values of 0–3, in jumps of 0.1) and the lapse rate parameter (ranging between the values of 0–0.05, in jumps of 0.01, see Eq 1). In total, these calculations result in 170,190 model simulations to cover all possible combinations for all participants. We then computed the log-likelihood for each model simulation. To account for the model’s additional parameters we calculated the corresponding AIC score and also used the log-likelihood ratio test (with and without a shutter). We assigned to each participant the scoring strategy with the best AIC score. For the section in the main text describing the differential weighting model, we added to the model for each participant the opponent’s weight parameter (ranging between the values of 0–0.5, in jumps of 0.1, see also Methods, Simulating a model with differential weighting of opponent’s moves). We note that we focused on the bias of missing ‘O’ paths to stay close to the behavioral findings indicating ‘O’ blindness.

### Measuring entropy of participants’ moves in the full and truncated boards

For a given board state in either the full or truncated board, we measured the entropy of participants’ moves by aggregating all participants’ moves (of that specific condition) to a distribution of probabilities over all open squares on the board. Next, we calculated the entropy of this distribution of moves per board configuration and condition (full vs. truncated). In the truncated board, we looked at the distribution of participants’ first moves. In the full board, we looked at the distribution of the third moves of only the fraction of participants that chose their first ‘X’ and ‘O’ moves such that they lead to the board state which the truncated configuration starts from. Thus, we compared entropy of the same board states reached from either the full or truncated boards.

### Simulating a model with internal search

It is possible that participants do some of the search internally, without reflecting their moves on the sandbox board. Then, they may re-use the results of these internal computations without these being reflected in the collected data. To examine the possible effects of internal search, we implemented two alternative models that utilize past computations. The first model uses the alpha-beta pruning algorithm, but assumes that at each turn, a search is performed up to a limited depth with the moves simulated in this first search are assumed not to be observed in the interface. The Alpha-beta pruning algorithm runs a depth-first search up to a certain depth, *d*, storing the best value the opponent can receive by using the scoring strategy to evaluate each node. This allows the algorithm to prune tree branches that would not be chosen by the opponent and thus to prune the search space. The scoring strategy also acts to order the moves to develop and to determine the value of the nodes at the maximal depth. Unlike MCTS, it does not store visitation counts but its memory is carried by the pruning of irrelevant paths. In the simulation, the selection of the next move was based on alpha-beta search with a limited depth (between depth of 1–3), such that the selection is based on a shallow search. This process is carried out iteratively for each chosen move, for either three moves on the full boards or one move for the truncated boards.

The second internal search model used Monte-Carlo Tree Search (MCTS) with a limited number of simulations (50, 200 or 500), assuming that participants only place an ‘X’ or an ‘O’ after doing these simulations internally, and re-use the results of their prior computations when examining the next move. That is, they retain the visit counts and the outcomes from previous MCTS simulations rather than starting anew at each move. We ran each model 100 times and examined the entropy of moves in the full and truncated boards. We note that this analysis is done only on the 6X6 boards since MCTS does not converge after 5,000 simulations on the 10X10 boards.

### Measuring path shutter in behavioral data

The path “shutter” size of each participant is the mean distance between participant’s moves and the potential paths induced by her last move. Specifically, we used the following procedure: If the chosen square belongs to one of the potential winning paths stemming from the last ‘X’ move, the distance is zero. Otherwise, the distance is the minimal Manhattan distance between the chosen square and the potential paths stemming from the previous ‘X’ move. A potential path is a valid path (horizontal, vertical or diagonal) of valid length (4 for 6x6 boards, 5 for 10x10 boards), which is not already blocked by the ‘O’ player. See S1 Text and S14 Fig for more details.

### Simulating the shutter heuristic’s effect on the probability to miss winning moves

To compute the expected effect of using a shutter heuristic on the probabilities of missing winning ‘O’ moves and winning ‘X’ moves, we performed the following procedure: we ran the alpha-beta algorithm with the “interaction” heuristic, but restricted the algorithm to consider only moves within a specified shutter size. For example, when setting a shutter size of 1, only squares that are on the potential paths of the previous ‘X’ move or at a Manhattan distance 1 from these paths are considered. We varied the shutter sizes, and made the search process non-deterministic by ranking the potential moves stochastically based on their scores (higher score is more likely to be chosen, proportional to the differences in scores). We then computed the effective shutter size in each simulation, and divided the simulations into narrow, medium, and wide shutter sizes using the values of the behavioral data. Finally, for each shutter category (narrow, medium, wide), we computed the likelihood of missing winning ‘X’ moves and the likelihood of missing winning ‘O’ moves.

### Simulating a model with differential weighting of opponent’s moves

An alternative model that could explain the observed “blindness” to the opponent’s winning moves is that participants pay less attention to their opponent positions than to their own. Different opponent’s weighting changes the weighting of the scores originating from preventing ‘O’ paths. These values range from 0.5 (i.e. equal contribution for gaining ‘X’ paths and preventing ‘O’ paths) to 0 (i.e. no contribution from preventing ‘O’ paths). To examine this, we considered variations to the scoring strategies computation by weighting the opponent’s threats (‘O’ potential winning sequences) differently. That is, the scoring strategy is:

$$\begin{array}{c} score_{pos}=\left(1-w_{opponent}\right)\cdot score_{pos}\left(X\right)+w_{opponent}\cdot score_{pos}\left(O\right) \end{array}$$ (2)

where *score*_*pos*_ is computed based on the scoring strategy (creating threats for ‘X’ and preventing ‘O’ threats) with values of *w*_*opponent*_ varying between completely ignoring the opponent (*w*_*opponent*_ = 0) and giving the opponent the same weight as for the paths of the ‘X’ player (default model, *w*_*opponent*_ = 0.5), in a resolution of 0.1 jumps (i.e., 0, 0.1, 0.2, 0.3, 0.4, 0.5).

### Computing the Pareto front for shutter values

To test the effects of a path shutter, we ran the alpha-beta pruning algorithm with an additional *shutter* parameter [values of 0, 0.5, 1 or 2]. This parameter determines which squares will be considered by the algorithm, and scores will only be computed for those squares. For shutter size zero, only squares on the potential paths of the last move are considered. For shutter size 1, only squares that are on the potential paths of the last ‘X’ move or at a Manhattan distance 1 from these paths are considered. When the shutter size is set to 0.5, squares at distance 1 will be considered with probability 0.5. In addition, we varied the following parameters—*Moves limit*: the maximal search size (30, 50, 100, and 200 moves). *Branching factor*: The number of nodes expanded in each level of the tree (3, 5, 7, and 10 nodes). *Score noise*: The standard deviation of the Gaussian noise added to the scores, *N*(0, *σ*) (*σ* ∈ [0, 2.5], increments of 0.25). This resulted in 1760 different configurations based on the possible value combinations of the parameters. We note that in this implementation the alpha-beta algorithm chooses which square to explore with probability which is in proportion to the square’s score and thus is not deterministic as the original alpha-beta algorithm. In total we ran 1760 configurations, each configuration 100 times. For each configuration, we measured the proportion of times that the algorithm was able to find the correct solution, and the required computational costs as measured by the number of squares for which a score had been computed. We then examined which shutter size values lie on the Pareto front in terms of accuracy and computation costs.

### Examining the effect of a path shutter on deep learning models

To further examine the effects of a shutter on computational systems, we trained deep reinforcement learning models to play the game. To this end, we used AlphaZero [66] (https://github.com/junxiaosong/AlphaZero_Gomoku), which learns through iterations of self-play, continuously improving its performance. The model consists of a policy-value network. The input to the network is the board state, and the network learns to predict the policy, i.e., probabilities for each possible action in a given state, and a value which represents the quality of the state. To represent the input board we use three 6X6 or 10X10 binary feature planes. The first two describe the positions of the ‘X’ and ‘O’ players on the board, and the third one is all ones or zeros indicating whose turn is it to play. The neural network consists of a “body” followed by both policy and value “heads”, similarly to AlphaGoZero architecture. The body consists of three 3X3 convolutional layers with padding of 1 and stride of 1, where the first layer uses 32 filters, the second uses 64 filters, and the last one uses 128 filters. We apply ReLU activation function after each convolutional layer. The policy head applies an additional 4-filter 1X1 convolutional layer with padding of 1 and stride of 1, followed by ReLU activation function. Finally, a softmax—linear layer of size 36 or 100 is applied. The value head also applies additional 2-filter 1X1 convolutional layer with padding of 1 and stride of 1, followed by ReLU activation function. The output is then passed through a softmax—linear layer of size 64 function, and finally a tanh—linear layer of size 1 is applied.

In each training epoch, the model is trained via self-play. The policy network guides the choice of moves which provides the prior probabilities for a Monte-Carlo Tree Search (MCTS). The loss function aims to match the values obtained in the games (for the value prediction) and the probabilities assigned by MCTS at the end of the epoch (for the policy prediction). We trained the models for 1500 training epochs. We trained a separate model for 6X6 boards and 10X10 boards, as their inputs are different. During training, each MCTS used 400 simulations. The learning rate was set to 0.002 and was adjusted to adapt at each step according to the KL divergence measure between the last step policy and the current policy. Moves were selected in proportion to the root visit count. Dirichlet noise (0.3) was added to the prior probabilities in the root node. Positions were batched across parallel training games for evaluation by the neural network. Adam optimizer was used. The L2 regularization parameter was set to 1e-4.

We then used the trained models to play against a strong vanilla MCTS algorithm which performed 1000 simulations at each turn on the boards used in our human experiments (S1 Text also includes games against an MCTS with 500 simulations). Specifically, we compared the performance of the trained model with that of the same model augmented with a shutter = 0 pruning heuristic—that is, a model which only considers moves that are within the shutter induced by the previous ‘X’ move. We tested several variations of the trained models (with/without the shutter), varying the number of MCTS simulations [0, 25, 50 or 100 simulations, see S1 Text for more details] that are done on top of the basic predictions of the network which serve as a prior. When adding the shutter heuristic, it is applied to the choice of moves in the game, as well as to the choice of moves in the MCTS simulations preceding the choice of a move. We note that we examine a relatively small number of simulations, since our hypothesis is that the shutter heuristic is useful when computation is limited. If a much larger number of simulations is used, we do not expect the shutter heuristic to help, as the MCTS algorithm can converge to the correct solution regardless.

## Supporting information

S1 Text Supporting information. **Table A**: Percent of boards solved with the alpha-beta algorithm in 35 nodes search by different scoring strategies.
(PDF)

S1 Fig Board configurations used in the experiments. Board I full: win within 4 moves, winning move: f3 or f5; Board I truncated: win within 3 moves, winning move: f5; Board II full: win within 4 moves, winning move: d6; Board II truncated: win within 3 moves, winning move: c6; Board III full: win within 5 moves, winning move: j6 or j9; Board III truncated: win within 4 moves, winning move: j9; Board IV full: win within 5 moves, winning move: e8; Board IV truncated: win within 4 moves, winning move: d8; Board V full: win within 5 moves, winning move: c6 or c7; Board V truncated: win within 4 moves, winning move: c5;.
(TIFF)

S2 Fig Participants’ actions do not exhibit correlation between thinking time prior to a “slow” action, and the number of subsequent “fast” actions that followed that move. **A)** Example histograms of the distribution of time between moves (in seconds) of 3 of the study participants, the plots only show moves taking up to 10 seconds of thinking. **B)** Scatter plot showing the time it took to execute a “slow” actions (x-axis) and the number of “fast” moves that followed the action. **C)** The distribution Spearman correlation values between thinking time prior to a “slow” action and the number of following “fast” moves among participants.
(TIFF)

S3 Fig Participants’ time on task and search size were not limited by time restriction. **A)** Participants’ solution times in each of the board configurations; **B)** Participants’ search size (number of actions) in each of the board configurations. **C)** Search size and solution time were highly correlated (Spearman correlation, r = 0.76, 95% CI = [0.73,0.78], *p* < 0.001). **D)** Search time and board complexity were not correlated (Spearman correlation, r = 0.02, 95% CI = [-0.05,0.08], *p* = 0.56).
(TIFF)

S4 Fig The number of moves explored by the alpha-beta algorithm using the different scoring strategies, and the number of moves tried by participants. (TIFF)

S5 Fig Log-likelihoods of participants first moves based on the predictions of the MCTS models. The log-likelihood is similar to that of a uniform prior. When adding the ‘Interaction’ scoring strategy to MCTS to prune possible moves (‘MCTS k = 5 n = 100’ and ‘MCTS k = 5 n = 5000’), the log-likelihood increases and is better than the uniform prior, but still significantly lower than that predicted by the scoring strategies alone.
(TIFF)

S6 Fig Heatmaps showing for each board configuration the distribution of participants’ first moves (behavioral data) and the predicted distribution according to the “Interaction” scoring strategy. (TIFF)

S7 Fig Percent of participants fitted to each scoring strategy. Left panel shows the distribution for the entire participant population; middle panel shows only participants who solved correctly; Right panel shows only participants who did not solve correctly.
(TIFF)

S8 Fig Percent of participants fitted to each scoring strategy in the “full” and “truncated” board conditions. (TIFF)

S9 Fig Sensitivity analysis for parameters used in the scoring strategy. **A)** Changes in log-likelihood fit for each scoring strategy when varying the winning score parameter; **B)** Changes in log-likelihood fit for the “Forcing” scoring strategy when varying the immediate threat score parameter. Note that this analysis considers the percent change in log-likelihoods of the scoring strategies, thus changes in the log-likelihoods do not have to be in opposite directions.
(TIFF)

S10 Fig Participants exhibit dependency of search moves on previous moves throughout the game. The entropy for equivalent board states encountered in the “full” condition is significantly lower than in the “truncated” condition. ***, *p* < 0.001.
(TIFF)

S11 Fig The differences in entropy between equivalent states in the “full” and “truncated” boards are not explained by differences in the population of participants (selection bias). **A)** Entropy in solvers’ moves when reaching equivalent game states in the “full” and “truncated” boards. **B)** Entropy in non-solvers’ moves when reaching equivalent game states in the “full” and “truncated” boards. **C)** When simulating moves of participants using their best fitting scoring strategy (without shutter) and filtering out simulations where the third move on the “full” board does not reach the first state of the “truncated” board, there is a small reduction in entropy (7%) but it does not explain the substantial reduction in entropy in the observational data. **D)** Simulations similar to (C), but with the scoring strategy augmented by the shutter, do explain the observed reduction in entropy in the “full” boards. ***, *p* < 0.001.
(TIFF)

S12 Fig A simulation of models using internal search (MCTS with 50, 200 or 500 simulations with memory of previous computations) do not show significant reduction in entropy between the move distribution in equivalent states of the truncated and full boards. Internal search using alpha-beta pruning with k = 7 and depth = 1 (the best fitted model) shows reduction in entropy, but not at the same extent as the behavioral data.
(TIFF)

S13 Fig MCTS search shows memory-less dynamics with similar entropy in the “Full” and “Truncated” conditions for equivalent states. (TIFF)

S14 Fig Example of square distances for shutter computation: if the last move was f5 (denoted with 1), there are three potential paths induced by this move, f6–f3, f5–f2 and c5–f5 (marked in blue). Squares on these paths are considered at distance 0 from the last move. Squares adjacent (Manhattan distance = 1) to squares on open paths are considered at distance 1 from the last move (marked in orange).
(TIFF)

S15 Fig Missed winning moves for the ‘X’ and ‘O’ player in computational simulations with a path shutter, and in the behavioral data. As the shutter size increases, the likelihood of missing winning ‘O’ moves reduces, while the likelihood of missing winning ‘X’ moves increases.
(TIFF)

S16 Fig The distribution of shutter values for participants’ moves that were not on a correct winning path, and the fraction of those moves that were optimal (optimal moves in orange, suboptimal moves in blue). (TIFF)

S17 Fig Log-likelihoods of scoring strategies’ predictions of participants’ moves. There are three configurations for each scoring strategy: using the scoring strategy as is (“base”), adding a shutter (based on best fit to participants), and further augmenting the scoring strategy with both a shutter and decreased attention to the opponent (based on best fit to participants). For all scoring strategies, adding the shutter significantly improved log-likelihood (in all cases *p* < 10^−5^ using a likelihood ratio test which accounts for the additional parameter), and except for the Density strategy (where opponent weight does not have a meaning), augmenting the scoring strategy with decreased attention to the opponent further increased the log-likelihood (2*p* < 10^−5^ using a likelihood ratio test which accounts for the additional parameter).
(TIFF)

S18 Fig Limiting the trained deep learning models with a shutter of size zero (blue bars) significantly improved their performance against an MCTS algorithm on all board configurations. A) Game win percentage of the deep learning models against MCTS with 1000 simulations; B) Game win percentage of the deep learning models against MCTS with 500 simulations. All differences were statistically significant with *p* < 10^−5^.
(TIFF)
