# Supplementary material for: Adaptive search space pruning in complex strategic problems
Source: PLoS Comput Biol. 2022 Aug 10;18(8):e1010358. doi: 10.1371/journal.pcbi.1010358 (PMC9394844; doi:10.1371/journal.pcbi.1010358)
Supplement: S1 Text — Table A: Percent of boards solved with the alpha-beta algorithm in 35 nodes search by different scoring strategies. (PDF) [file pcbi.1010358.s001.pdf]

Adaptive Search Space Pruning in Complex Strategic Problems: Supporting Information

Ofra Amir[1], Liron Tyomkin[1], Yuval Hart[2]

1 Faculty of Industrial Engineering and Management, Technion - Israel Institute of Technology, Haifa, Israel
2 Department of Psychology, Hebrew University of Jerusalem, Jerusalem, Israel

S1 Experimental Paradigm

We presented participants with a "k-in-a-row" game with different board configurations which required finding a move for the 'X' player that will force a win within a given number of turns.[1] We constructed five board configurations which differed in difficulty. Two of the configurations were 6x6 boards with a '4 in a row' winning rule, and three of the configurations were 10x10 boards with a '5 in a row' winning rule. For each of these basic five board configurations, we generated an additional board in which we showed participants a *truncated* version of that board, with an additional marks of the optimal move for 'X' and 'O' from the initial board. Thus, in these *truncated* configurations, participants had to indicate the move that will force a win within either 3 or 4 turns (corresponding to the conditions with 4 or 5 turns in the *full* version respectively). In total, the experiment included ten conditions, where in each condition one of the five board configurations was presented, in either its "full" or "truncated" version (S1 Fig).

S2 Participants' moves on the board reflect their search process

Since participants are not constantly active on the 'sandbox' board, in this section we analyze participants' behavior on and off the provided 'sandbox' to infer what is captured on the tracked search trajectory of participants. One possibility for participants to use the sandbox is to mentally search the tree of options in their mind, and only then execute a set of pre-determined actions they. To check this possibility, we analyzed the time taken between actions - We classify participants' actions into two categories: 1. "slow" actions - actions preceded by relatively long duration of thinking (over 2.5 seconds from the previous moves) and 2. "automatic" actions that could reflect simple execution of a planned path (less than 2.5 seconds from the previous move). We chose the 2.5 seconds threshold by examining the empirical distribution of thinking times before participants take actions (see examples in S2A Fig).

We then checked whether there is a correlation between the time participants took to think before executing a "slow" action, and the number of subsequent "automatic" actions that followed the thinking period. If participants were simply executing a planned path, we would expect to find a positive correlation between the two. However, we find a very small negative correlation value (Spearman correlation, r=-0.08, 95% CI=[-0.11,-0.06], $p < 0.001$, see S2B Fig), indicating that there is no positive relation

[1]The experiment can be run online here: `http://ec2-54-149-86-189.us-west-2.compute.amazonaws.com/webTTT/public_html/tictactoe/tictactoe.html?board=1f`

between the time participants take to think of a move and subsequent moves they carry. We further analyzed these correlations for each participant separately, and find that the majority of the participants do not exhibit a positive correlation, and that there is no clear pattern of correlation between thinking time and "automatic" actions, see S2C Fig.

Furthermore, we find that the mean number of "automatic" moves after any slow action period is 0.63 (95% CI=[0.6, 0.66]), and the median time between moves is 2.8 seconds (95% CI=[2.76, 2.9]), both indicating that the participants interact continuously with the sandbox board and consequently that their moves reflect well the search process they are going through.

We note that these results do not contradict any internal search before using the sandbox to find the winning move, for example it may be that there are generally fast participants and generally slow participants.

S3 Participants' solution time was not restricted by the allotted time to solve

Participants' solution time was substantially smaller than the allotted 10 minutes (Median work time= 3.87 minutes, Min-Max: 0.32-10 minutes, see S3A Fig. We note that participants could submit their solution before the 10 minutes expired. Moreover, participants' search size and solution time were highly correlated (Spearman correlation, r= 0.76, 95% CI=[0.73,0.78], $p < 0.001$; S3 Fig), suggesting that their search was not limited due to time constraints. In addition, there was no correlation between board complexity and search time (Spearman correlation, r= 0.02, 95% CI=[-0.05,0.08], $p = 0.56$, S3D Fig).

S4 Complete description of the scoring strategies

We used the following scoring strategies to evaluate the quality of moves:

1. *Density*: Score of each square is determined by the number of neighbors already marked by 'X'. We used a neighborhood size of 2, where each neighboring 'X' that is adjacent to the square contributes 1/8 (as there are 8 adjacent squares), and each 'X' in a radius of 2 squares contributes 1/16 to the final score. We note that using a different neighborhood size results in similar qualitative results.

2. *Linear*: Score of each square is determined by the sum score of potential 'winning paths' it is on. Each path score is the number of squares filled by 'X' (including the potential square for which the score is calculated). For example, a square which belongs to two different paths, one path with 2 'X' squares and one path with 3 'X' squares will receive a score of 2+3=5. The square receives an additional score for blocking potential opponent winning paths (similar computation for path score, but not counting the currently examined square as it is not the opponent's turn).

3. *Non-linear*: Score of each square is determined by the sum score of potential 'winning paths' they are part of. Each path contributes a non-linear score $S_{non-linear} = \frac{1}{n-n_i}$ (where n is the target number of 'X' for a win, and n_i is the number of 'X' currently in path i). For example, a square which belongs to two different paths, one path with 2 'X' squares and one path with 3 'X' squares (when the target is '5 in a row') will receive a score of $S_{non-linear} = \frac{1}{5-2} + \frac{1}{5-3} = 5/6$. Thus, the closer the path is to '5 in a row', the

score increases substantially. Additional score is given for blocking opponent's paths using the same computation.

4. *Interaction*: Score of each square is determined by the sum score of potential 'winning paths' they are part of and the interaction between them. The score is based on the non-linear scoring strategy augmented by interaction terms from paths that share potential squares (but that cannot be blocked simultaneously by a single 'O' move). The added score is $S_{interaction} = S_{non-linear} + \frac{n_i \cdot n_j}{(n-1)^2 - n_i \cdot n_j}$. For example, a square which belongs to two different paths, one path with 2 'X' squares and one path with 3 'X' squares will receive an additional score of $\frac{2 \cdot 3}{(5-1)^2 - (2 \cdot 3)} = 0.6$. Additional score is given for blocking opponent paths using the same computation.

5. *Forcing*: This scoring is similar to *interaction*, but includes an additional component: if placing an 'X' in the square results in an immediate threat (a path that now contains $n - 1$ 'X' markers with a potential to win in the next move), the score of the square is augmented by a large constant (10 points) since by forcing 'O' to a particular move, 'X' essentially blocked all other paths for 'O' and controls the game flow.

We note that because the algorithm compares squares' scores to decide which square to choose next, it is the relative scores between different squares that is important rather than their absolute value.

S5 Alpha-beta algorithm accuracy depends on the scoring strategies

The search size required by the alpha-beta pruning algorithm for optimally solving the different board configurations highly depends on the scoring strategies used (S4 Fig).

When using sophisticated scoring strategies to evaluate moves, the alpha-beta algorithm can reach the optimal solution even with a search size limited to that exhibited by participants (Table A). We note that in this case the alpha-beta search is not guaranteed to return the correct solution as not all moves are examined, but the algorithm still returns the best solution it found in its search.

S6 Monte-Carlo Tree Search improves with sophisticated scoring strategies

We compared participants' search with the Monte-Carlo Tree Search (MCTS) algorithm with an Upper Confidence bound applied to Trees (UCT) exploration policy. MCTS randomly searches the tree of possible moves and performs "rollouts" (simulations), i.e. executes a random trajectory until reaching a terminal state to assess the likelihood of winning from each node. We ran four different configurations of MCTS, varying the number of nodes that are explored ($n = 100$ or $n = 5000$) and varying whether MCTS explores all possible moves in each board state ("All") or MCTS explores only the top 5 moves (as ranked by the 'Interaction' scoring strategy ($k = 5$)). Thus, the four search configurations were 'MCTS All, n=100', 'MCTS All, n=5000', 'MCTS k=5, n=100' and 'MCTS k=5, n=5000'.

To assess the resemblance of participants' moves to the different MCTS configurations, we computed the log-likelihood of participants' first moves based on the predictions of the different MCTS configurations. We find that the log-likelihood values

are significantly higher when pruning the space using the Interaction scoring strategy compared to uninformed search (see S5 Fig). We note, however, that even with the additional pruning, the MCTS models do not explain well participants' behavior (low log-likelihood values).

S7 Fit of scoring strategies to participants' behavior

The scoring strategies can be used to assign a predicted probability distribution over the board positions (see S6 Fig for a comparison between the first move distribution in the behavioral data and the distribution predicted by the "Interaction" scoring strategy). In addition to the log-likelihood analysis of the scoring strategies described in the main text, we also calculated the percent of participants in the population that employ each scoring strategy, and the log-likelihood of the fitting process of each sub-population. To this end, we fitted to each participant the scoring strategy that best match their moves: First, for each scoring strategy, we computed a probability distribution over moves for each game state. The probability of placing an 'X' or an 'O' in a particular square s using a scoring strategy h is computed by dividing each square score with the total scores over all squares on the board. Then, we calculated the log-likelihood and respective AIC values for each scoring strategy, and fitted to each participant the scoring strategy with the lowest AIC value. Having a scoring strategy fit for each participant, we calculated the percent of participants in the population that employ each scoring strategy. We find that for about half of the participants, the "Interaction" and "Forcing" scoring strategies provided a better fit (51% of the population). A minority of participants were using the simpler scoring strategies, termed "Density" and "Linear" (Percent of participants, Density: mean=8% , 95% CI=[6%, 9%], Linear: mean=6% , 95% CI=[5%, 8%], Non-Linear: mean=28% , 95% CI=[25%, 31%], Interaction: mean=35% , 95% CI=[32%, 38%], Forcing: mean=23%, 95% CI=[20%, 25%])

In addition, we analyzed the distribution of the scoring strategies for the solvers and non-solvers populations separately, and observed that there are more participants who use sophisticated scoring strategies in the solvers population compared to the non-solvers population (see S7 Fig).

Lastly, to ensure that the differences between the distributions of moves in the "full" and "truncated" boards are not a result of participants deploying different strategies, we compared the distribution of scoring strategies in the different conditions. As can be seen in S8 Fig, there are not very substantial differences in the scoring strategies used in the two conditions.

S8 Sensitivity analysis of the scoring strategies' fit

All scoring strategies use a parameter that sets the score of a winning move. This score should approximately be infinity since the player wins the game, yet in the simulations it needs to have a finite value. If a winning square exists, the winning score normalizes all other scores on the board, and defines the probability of missing a winning move for 'X'. The "Forcing" scoring strategy has an additional parameter that defines the additional bonus score given for creating an immediate threat. We analyzed the sensitivity of the scoring strategies fit to participants' moves by varying these parameters in a range of up to 100 folds (win scores between 25–2500, immediate threat scores between 2–200) and assessing the log likelihood fit of each scoring strategy to each of the participants.

When varying the score for winning the game by up to 100 folds, we find changes of only up to 20% in the values of the log-likelihood fit (S9A Fig). Similarly, when varying the score for creating an immediate threat on the opponent by up to 100 folds, we find

changes of up 13% (note this change only affects the "Forcing" scoring strategy, S9B Fig).

S9 Participants search trajectories indicate a dependency of the current moves on previous moves

In the main text we presented comparisons between the entropy of the "full" and "truncated" boards in participants' first moves. Here, we present a similar calculation, this time accounting for all similar boards configurations, even if they occur at later times in the game. When examining the entropy of participants' moves in equivalent states encountered in the "full" and "truncated" conditions throughout the game, we find that the entropy in the "full" condition is significantly lower than in the "truncated" condition (full: mean=0.58, 95% CI=[0.52, 0.65], truncated: mean=0.96, 95% CI=[0.89, 1.03], full vs. truncated: Mann-Whitney test U=0, $p < 0.001$, Rank biserial correlation=1.0; see S10 Fig).

S10 Selection bias does not explain the entropy differences between the full and truncated boards

One possible explanation for the observed differences in the entropy for moves in equivalent states in the "full" and "truncated" boards could be differences in the population of participants that reached those states. A hypothetical case could be if in the full board, less noisy participants found the optimal first move, while on the truncated board - all participants see the starting board state which assumes the first two optimal moves from the "full" board. To ensure that this was not the case, we conducted two analyses. First, we examined the entropy of moves separately for solvers and non-solvers. We found that for both populations there is significantly higher entropy in the "truncated" board configurations (Solvers: full: mean=0.34, 95% CI=[0.27, 0.41], truncated: mean=0.68, 95% CI=[0.6, 0.77], full vs. truncated: Mann-Whitney test U=0, $p < 0.001$, Rank biserial correlation=1.0; Non-solvers: full: mean=0.73, 95% CI=[0.65, 0.82], truncated: mean=1.2, 95% CI=[1.1, 1.28], full vs. truncated: Mann-Whitney test U=0, $p < 0.001$, Rank biserial correlation=1.0; see S11 Fig).

Second, we considered a model where noisy participants are more likely to not be observed in the "full" board states that are equivalent to the "trunacated" board initial states. To this end, we simulated moves for each participant using its best fitting scoring strategy, once without a shutter, and once with a shutter. We repeat this process 100 times for each participant and for each initial board configuration, and filter out simulations from the "full" boards that do not reach the board state that is equivalent to the corresponding "truncated" board configuration. This process naturally filters out noisy participants, as they are less likely to find the first optimal moves, and hence are less likely to reach the board states equivalent to the "trauncated" board configurations. When running the simulations with the base models (best fitting scoring strategy without shutter), we find a significant yet very small ($\approx 7\%$) reduction in entropy which does not resemble the large reduction in entropy observed in the behavioral data (full: mean = 3.23, 95% CI=[3.23, 3.23], truncated: 3.47, 95% CI=[3.47, 3.47], $p < 0.001$, Rank biserial correlation=1.0). When augmenting the scoring strategies with the shutter, we do observe a significant and substantial reduction in entropy, similar to that found in the behavioral data (full: mean = 2.47, 95% CI=[2.47, 2.47], truncated: 3.48, 95% CI=[3.48, 3.48], $p < 0.001$, Rank biserial correlation=1.0).

S11 Models using internal search or opponent weighting do not explain the reduction in entropy between the full and truncated boards

Though our paradigm records much more of the search trajectories of participants, it is still quite possible that participants do some of the search internally in their mind, without reflecting their moves on the sandbox board. Then, they may re-use the results of these internal computations without these being reflected in the collected data. To examine the effect of such a model, we run Monte-Carlo Tree Search (MCTS) with a limited number of simulations (50, 200 or 500), assuming that participants only place an 'X' or an 'O' after doing these simulations internally, and re-use the results of their prior computations when examining the tree for their next move. That is, they retain the visit counts and the outcomes from previous MCTS simulations rather than starting anew at each move. We ran each model 100 times and aggregated the statistics to examine the entropy of moves in the full and truncated boards. We note that this analysis is done only on the 6X6 boards since the MCTS algorithm does not converge without running a much greater number of simulations. We find that the internal search model does not produce the differences in entropy between move distributions in equivalent board states reached from the truncated and full boards (MCTS with 50 simulations: full: mean=2.53, 95% CI=[2.45, 2.61], truncated: mean=2.65, 95% CI=[2.57, 2.72], MCTS with 200 simulations: full: mean=2.25, 95% CI=[2.05, 2.44], truncated: mean=2.38, 95% CI=[2.22, 2.55, MCTS with 500 simulations: full: mean=1.44, 95% CI=[1.01,1.93], truncated: mean=1.6, 95% CI=[1.24, 1.99], see S12 Fig).

We further implemented a second model of internal search that uses alpha-beta pruning rather than MCTS. In this model, at each turn, a search is performed up to a limited depth. The moves simulated in this first search are assumed not to be observed in the interface. Based on this search, the next move on the board is chosen, and the the process repeats. This model has two parameters: d is the depth of the shallow (unobserved) search, k is the number of nodes considered at each level of the tree (values between 5–10). This model does not directly "store" previous computations, but it might show a dependence in previous moves due to the "internal" search focusing on some parts of the tree. This model does predict a lower entropy in the truncated boards (entropy in the best fitting model, full: mean=1.51, 95% CI=[1.4, 1.61], truncated: mean=1.83, 95% CI=[1.73, 1.92], S12 Fig). However, the reduction in entropy is lower than that observed in the behavioral data. In particular, while the entropy value in the full boards is similar to that observed in the behavioral data, the entropy in the truncated boards is lower (1.83 for internal search vs. 2.42 in the behavioral data), possibly because of the added internal search. We also computed the log-likelihood fit of this model to participants' moves and find that the fit is substantially worse than that of the shutter model (log-likelihood of best fitting alpha-beta internal search model: -3.17, 95% CI=[-3.24, -3.09] compared with a log-likelihood of -2.21, 95% CI [-2.17, -2.25] for the "Forcing" augmented with a shutter model). We note that the log-likelihood fit for the first moves was similar to that of the best fitting scoring strategies, as this model used the "Forcing" strategy. However, the likelihood for more advanced moves was substantially lower, suggesting that the model does not provide a good fit to the behavioral data.

Since participants exhibited "blindness" to their opponent moves, we also examined a model that assigns a lower weight to blocking the opponent's potential winning moves (see Methods). As shown in the main manuscript, this model does not exhibit any reduction in entropy and as such does not fully explain the behavioral data. We further tested the log-likelihood fit of this model. As shown in S17 Fig, this model does improve the log-likelihood fit of most base scoring strategies, however, it does not

provide a better fit than the shutter heuristic (the differences between a model with a shutter and a model with reduced opponent weight were not significant for the Forcing and Interaction scoring strategies, and in all other cases the shutter heuristic provided a significantly better fit). We note that augmenting the scoring strategies with both heuristics (the shutter and opponent weighting) increased the log-likelihood but did not significantly improve the AIC values when accounting for the additional parameter ($p > 0.05$ for all scoring strategies). We also tested the mean log-likelihoods when assigning to each participant their best fitting model. That is, for each participant, we take the best fitting scoring strategies, and assess its fit when using the base scoring strategy alone, the scoring strategy augmented by the optimal shutter for that participant, or the scoring strategy augmented by the optimal opponent weighting heuristic for that participant. We find a similar improvement to the log-likelihood when using either the shutter heuristic or the opponent weighting (mean log-likelihood base models: -2.5, mean log-likelihood shutter models: -2.12, mean log-likelihood opponent weighting models: -2.15).

S12 MCTS exhibits memory-less search dynamics

MCTS is a Markovian algorithm and thus the decision about which move to make in a particular board state does not depend on how that state was reached but only on the current board configuration. This can be seen empirically when comparing the entropy of the first board state in the truncated configurations with the equivalent third board state in the full configuration (full: mean entropy=1.9, 95% CI=[0.5, 2.8], truncated: mean entropy=1.9, 95% CI=[0.3, 3.0], Mann-Whitney test U=12466017, $p = 0.4$, Rank biserial correlation=0.003, S13A Fig). We note that alpha-beta pruning is also Markovian, since in every board configuration it chooses the moves according to their scoring strategies and thus it does not take into account any past actions.

S13 Shutter computation for behavioral data

The “shutter” size of each participant is the mean distance between the participant’s chosen moves and the potential paths of her previous move. Specifically, we used the following procedure: If the chosen square belongs to one of the potential paths stemming from the previous ‘X’ move, the distance is zero. Otherwise, the distance is the minimal Manhattan distance between the chosen square and the potential paths stemming from the previous ‘X’ move (see S14 Fig for an illustration). A potential path is a valid path (horizontal, vertical or diagonal) of valid length (4 squares for 6x6 boards, 5 squares for 10x10 boards), which is not already blocked by the ‘O’ player.

S14 The use of a path shutter predicts the “blindness” to winning ’O’ moves

To infer the effects of a shutter mechanism on the probability to miss either ‘X’ or ‘O’ winning moves, we simulated the search while varying the available squares for the next move - The wider the shutter, the less constrained is the search to the squares on the potential ‘X’ paths to win (see Methods for more details). The simulated shutter model indicates that as the shutter size grows wider, the likelihood of missing ‘O’ winning moves reduces and the likelihood of missing ‘X’ moves increases, making the two likelihoods closer to each other (Simulations, Median ratio of missed ‘X’ wins to missed ‘O’ wins: narrow shutter: median= 0.05, 95% CI =[0, 0.16], medium shutter:

median=0.45, 95% CI =[0.38, 0.53], wide shutter: median= 0.67, 95% CI =[0.62, 0.73], Mann-Whitney tests: narrow vs. medium: U=72453, $p < 0.001$, Rank biserial correlation=0.28, narrow vs. wide: U=153697, $p < 0.001$, Rank biserial correlation=0.38, medium vs. wide: U=332656, $p < 0.001$, Rank biserial correlation=0.13). Thus, the simulated model predicts that the ratio of missed 'X' wins to missed 'O' wins should grow with shutter size.

Indeed, we find similar results in the behavioral data - As participants' shutter size increases, the ratio between missed 'X' wins to missed 'O' wins grows as well (Participants, Median ratio of missed 'X' wins to missed 'O' wins: narrow shutter: median= 0, 95% CI =[0, 0], medium shutter: median=0.33, 95% CI =[0.27, 0.42], wide shutter: median= 0.5, 95% CI =[0.44, 0.67], Mann-Whitney tests: narrow vs. medium: U=11523, $p < 0.001$, Rank biserial correlation=0.3, narrow vs. wide: U=10034, $p < 0.001$, Rank biserial correlation=0.38, medium vs. wide: U=23201.5, $p = 0.0007$, Rank biserial correlation=0.17). The increase in the ratio of missed wins as shutter size increases is driven by both a significant increase in missed 'X' wins and a significant decrease in missed 'O' wins (See S15 Fig).

S15 Participants are likely to make narrow-shutter moves even when they are sub-optimal

One might claim that the observed shutter mechanism is a by-product of participants choices of optimal moves. To address this, we explored whether people make moves within a narrow shutter simply because these are the optimal moves when trying to win the game (building on prior threats) or does the narrow shutter appear also on sub-optimal paths. To this end, we examined the shutter values of moves that follow a prior move that was not along the winning path, and further examined whether given the current board state, the moves were optimal or not. We find that the majority of moves (65%, S16 Fig) are within a zero shutter distance from their previous move. Moreover, of those moves, only 50% were the optimal move given the current state. These results suggest that people follow the shutter mechanism even when it leads to suboptimal outcomes, as was also seen in the analysis of blindness to winning 'O' moves.

S16 Adding a shutter mechanism and reduced attention to opponent increases model fit to behavioral data

We computed the log-likelihood of participants' moves based on the predictions made by the different scoring strategies, augmented with a shutter mechanism (shutter = 0) and with reduced attention to the opponent's moves (i.e., not considering opponent threats in the computation of the scoring strategies, see Methods). We find that adding both mechanisms improves the log-likelihood (see **??** Fig). In all cases, adding the shutter mechanism increased the log-likelihood (see main text). In addition, for all scoring strategies except Density (which does not take into account the opponent's pieces in the first place), augmenting the model with a reduced weight for attention to opponent moves further increases the log-likelihood values ($p <= 0.0001$ for all comparisons of the scoring strategy with shutter and reduced focus on opponent vs. the scoring strategy with only the shutter mechanism).

S17 The use of a path shutter improves the performance of deep learning models under computational restrictions on search

To further evaluate the effect of using a path shutter mechanism on computational methods, we trained a deep learning model (see Methods) to play the game and tested its performance with and without the addition of a shutter mechanism. To this end, we had each model (with/without shutter limitation) play 1000 times against two configurations of a Monte Carlo Tree Search (MCTS) algorithms, one using 500 simulations to evaluate each move, and the other using 1000 simulations to evaluate each move. The shutter mechanism was used to prune the moves considered by the model with a shutter value of zero, that is, only moves along threats created in the previous move were considered.

As can be seen in S18 Fig, adding the shutter limitation as a pruning mechanism significantly and substantially improved the performance of the model against MCTS with 1000 simulations (Board I full wins ratio: No shutter: 0.011, 95% CI= [0.004, 0.015], With shutter: 0.065, 95% CI= [0.050, 0.081], Board I truncated wins ratio: No shutter: 0.152, 95% CI= [0.129, 0.174], With shutter: 0.403, 95% CI= [0.373, 0.433], Board II full wins ratio: No shutter: 0.012, 95% CI= [0.006, 0.019], With shutter: 0.144, 95% CI= [0.123, 0.166], Board II truncated wins ratio: No shutter:0.047, 95% CI= [0.034 0.061], With shutter: 0.159, 95% CI= [0.137 0.182], Board III full wins ratio: No shutter: 0.001, 95% CI= [0, 0.003], With shutter: 0.259, 95% CI= [0.232, 0.286], Board III truncated wins ratio: No shutter: 0.0, 95% CI= [0, 0], With shutter: 0.113, 95% CI= [0.094 0.133], Board IV full wins ratio: No shutter: 0.027, 95% CI= [0.017, 0.037], With shutter: 0.191, 95% CI= [0.167, 0.216], Board IV truncated wins ratio: No shutter: 0.023, 95% CI= [0.014 0.033], With shutter: 0.188, 95% CI= [0.164 0.212], Board V full wins ratio: No shutter: 0.216, 95% CI= [0.191, 0.242], With shutter: 0.429, 95% CI= [0.398, 0.460], Board V truncated wins ratio: No shutter: 0.05, 95% CI= [0.037 0.064], With shutter: 0.24, 95% CI= [0.214 0.267], all $p < 10^{-5}$.). We also show the performance of the model with and without the shutter on full games (empty 6X6 or empty 10X10 boards), where an improvement is shown in both, but more substantially in the 6X6 board configuration.

Similar results were obtained when playing against MCTS with 500 simulations (Board I full wins ratio: No shutter: 0.013, 95% CI= [0.004, 0.017], With shutter: 0.069, 95% CI= [0.054, 0.085], Board I truncated wins ratio: No shutter: 0.148, 95% CI= [0.125, 0.168], With shutter: 0.421, 95% CI= [0.391, 0.452], Board II full wins ratio: No shutter: 0.083, 95% CI= [0.066, 0.1], With shutter: 0.320, 95% CI= [0.291, 0.349], Board II truncated wins ratio: No shutter: 0.206, 95% CI= [0.181, 0.231], With shutter: 0.423, 95% CI= [0.392, 0.454], Board III full wins ratio: No shutter: 0.0, 95% CI= [0., 0.], With shutter: 0.338, 95% CI= [0.309, 0.368], Board III truncated wins ratio: No shutter: 0.002, 95% CI= [0., 0.005], With shutter: 0.142, 95% CI= [0.121, 0.164], Board IV full wins ratio: No shutter: 0.061, 95% CI= [0.047, 0.076], With shutter: 0.231, 95% CI= [0.205, 0.258], Board IV truncated wins ratio: No shutter: 0.049, 95% CI= [0.036, 0.063], With shutter: 0.271, 95% CI= [0.244, 0.299], Board V full wins ratio: No shutter: 0.36, 95% CI= [0.33, 0.39], With shutter: 0.573, 95% CI= [0.542, 0.604], Board V truncated wins ratio: No shutter: 0.083, 95% CI= [0.066, 0.1], With shutter: 0.329, 95% CI= [0.3, 0.358], all $p < 10^{-5}$).

Tables

Scoring strategy	Boards solved (35 nodes)
Density	1/10
Linear	2/10
Interaction	5/10
Non-linear	6/10
Forcing	9/10

Table A. The number of boards for which the alpha-beta algorithm reached the correct solution with search limited to 35 nodes, using each of the different scoring strategies. Sophisticated scoring strategies enable the algorithm to find the correct solution with very limited search size.
