# Supplementary figures and images for: Adaptive search space pruning in complex strategic problems
Source: PLoS Comput Biol. 2022 Aug 10;18(8):e1010358. doi: 10.1371/journal.pcbi.1010358 (PMC9394844; doi:10.1371/journal.pcbi.1010358)

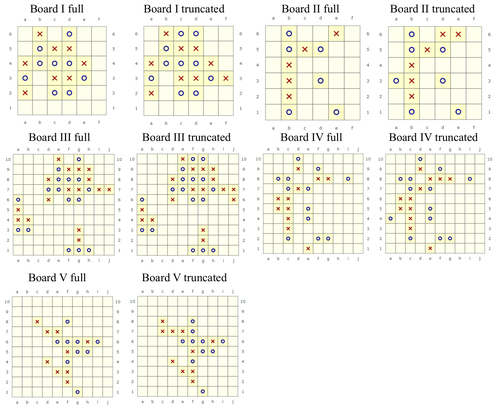

Supplement: S1 Fig — Board I full: win within 4 moves, winning move: f3 or f5; Board I truncated: win within 3 moves, winning move: f5; Board II full: win within 4 moves, winning move: d6; Board II truncated: win within 3 moves, winning move: c6; Board III full: win within 5 moves, winning move: j6 or j9; Board III truncated: win within 4 moves, winning move: j9; Board IV full: win within 5 moves, winning move: e8; Board IV truncated: win within 4 moves, winning move: d8; Board V full: win within 5 moves, winning move: c6 or c7; Board V truncated: win within 4 moves, winning move: c5;. (TIFF) [file pcbi.1010358.s002.tiff]

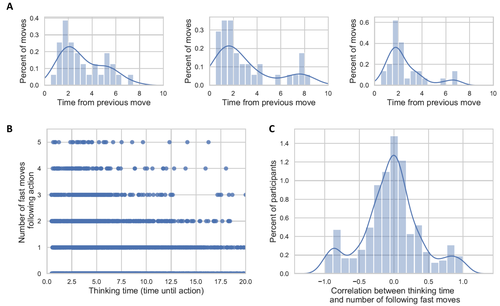

Supplement: S2 Fig — A) Example histograms of the distribution of time between moves (in seconds) of 3 of the study participants, the plots only show moves taking up to 10 seconds of thinking. B) Scatter plot showing the time it took to execute a “slow” actions (x-axis) and the number of “fast” moves that followed the action. C) The distribution Spearman correlation values between thinking time prior to a “slow” action and the number of following “fast” moves among participants. (TIFF) [file pcbi.1010358.s003.tiff]

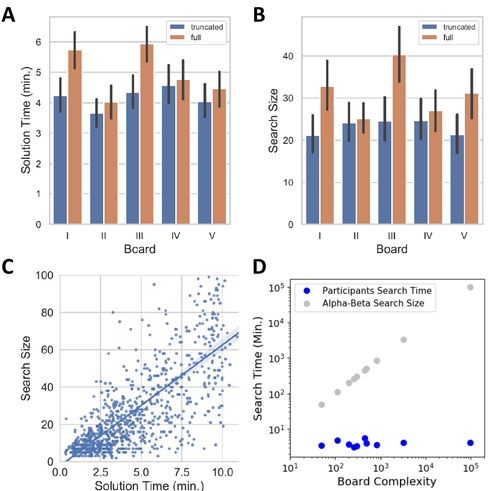

Supplement: S3 Fig — A) Participants’ solution times in each of the board configurations; B) Participants’ search size (number of actions) in each of the board configurations. C) Search size and solution time were highly correlated (Spearman correlation, r = 0.76, 95% CI = [0.73,0.78], p < 0.001). D) Search time and board complexity were not correlated (Spearman correlation, r = 0.02, 95% CI = [-0.05,0.08], p = 0.56). (TIFF) [file pcbi.1010358.s004.tiff]

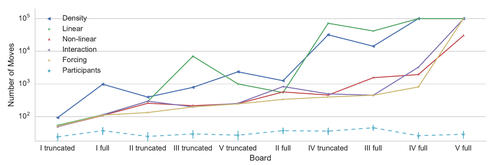

Supplement: S4 Fig — (TIFF) [file pcbi.1010358.s005.tiff]

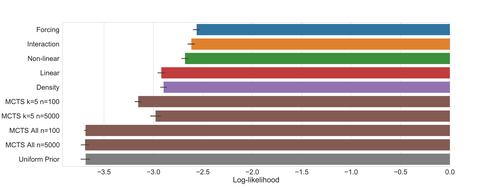

Supplement: S5 Fig — The log-likelihood is similar to that of a uniform prior. When adding the ‘Interaction’ scoring strategy to MCTS to prune possible moves (‘MCTS k = 5 n = 100’ and ‘MCTS k = 5 n = 5000’), the log-likelihood increases and is better than the uniform prior, but still significantly lower than that predicted by the scoring strategies alone. (TIFF) [file pcbi.1010358.s006.tiff]

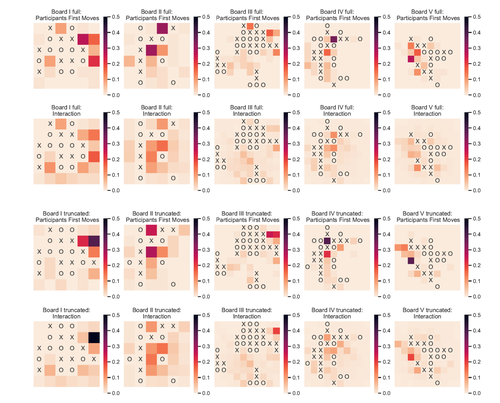

Supplement: S6 Fig — (TIFF) [file pcbi.1010358.s007.tiff]

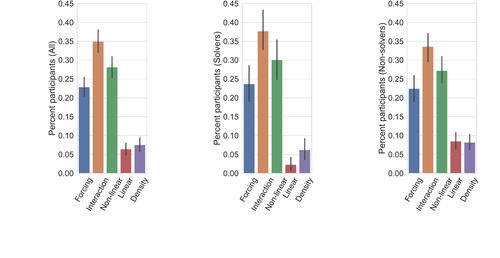

Supplement: S7 Fig — Left panel shows the distribution for the entire participant population; middle panel shows only participants who solved correctly; Right panel shows only participants who did not solve correctly. (TIFF) [file pcbi.1010358.s008.tiff]

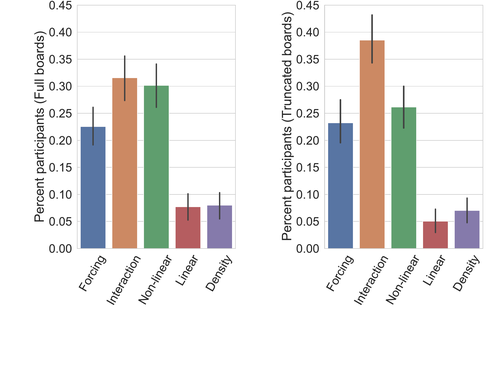

Supplement: S8 Fig — (TIFF) [file pcbi.1010358.s009.tiff]

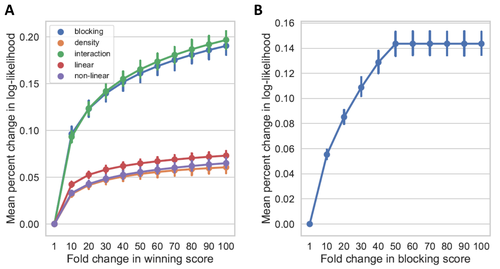

Supplement: S9 Fig — A) Changes in log-likelihood fit for each scoring strategy when varying the winning score parameter; B) Changes in log-likelihood fit for the “Forcing” scoring strategy when varying the immediate threat score parameter. Note that this analysis considers the percent change in log-likelihoods of the scoring strategies, thus changes in the log-likelihoods do not have to be in opposite directions. (TIFF) [file pcbi.1010358.s010.tiff]

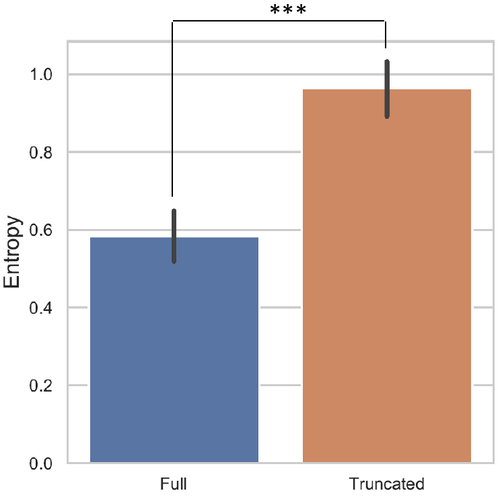

Supplement: S10 Fig — The entropy for equivalent board states encountered in the “full” condition is significantly lower than in the “truncated” condition. ***, p < 0.001. (TIFF) [file pcbi.1010358.s011.tiff]

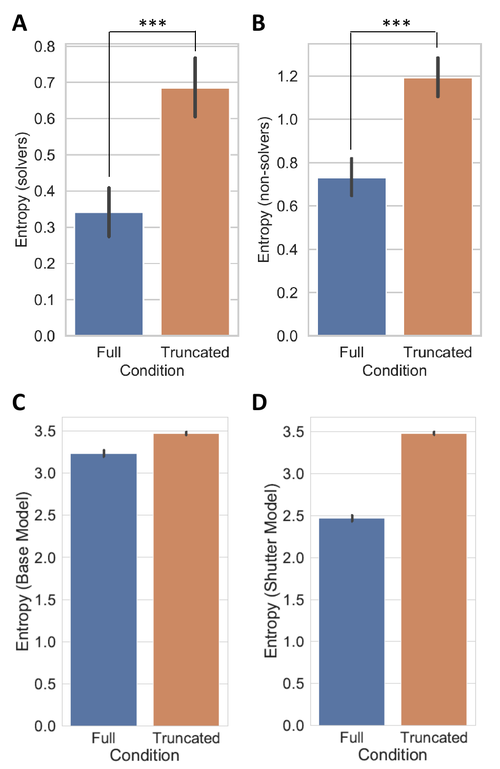

Supplement: S11 Fig — A) Entropy in solvers’ moves when reaching equivalent game states in the “full” and “truncated” boards. B) Entropy in non-solvers’ moves when reaching equivalent game states in the “full” and “truncated” boards. C) When simulating moves of participants using their best fitting scoring strategy (without shutter) and filtering out simulations where the third move on the “full” board does not reach the first state of the “truncated” board, there is a small reduction in entropy (7%) but it does not explain the substantial reduction in entropy in the observational data. D) Simulations similar to (C), but with the scoring strategy augmented by the shutter, do explain the observed reduction in entropy in the “full” boards. ***, p < 0.001. (TIFF) [file pcbi.1010358.s012.tiff]

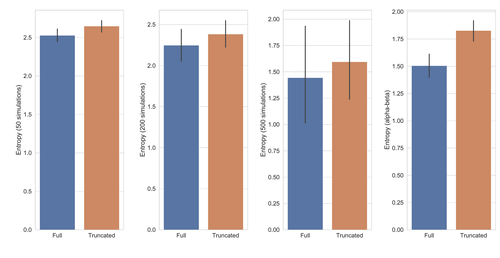

Supplement: S12 Fig — Internal search using alpha-beta pruning with k = 7 and depth = 1 (the best fitted model) shows reduction in entropy, but not at the same extent as the behavioral data. (TIFF) [file pcbi.1010358.s013.tiff]

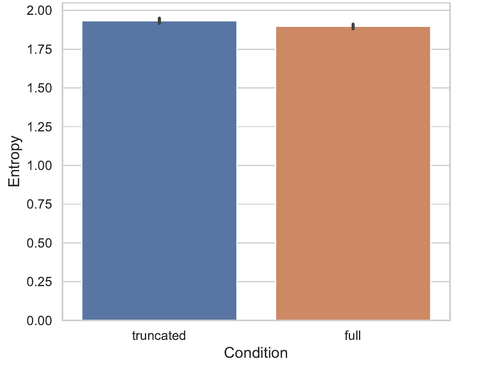

Supplement: S13 Fig — (TIFF) [file pcbi.1010358.s014.tiff]

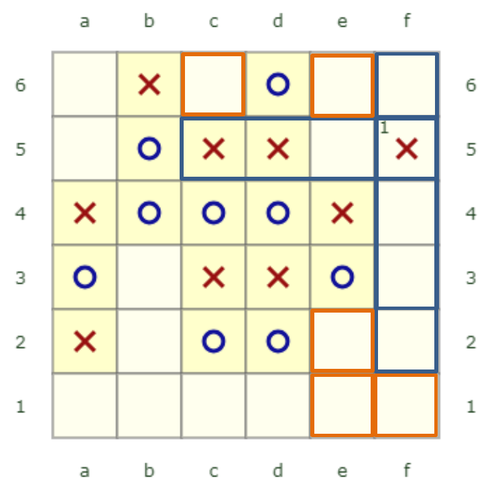

Supplement: S14 Fig — Squares on these paths are considered at distance 0 from the last move. Squares adjacent (Manhattan distance = 1) to squares on open paths are considered at distance 1 from the last move (marked in orange). (TIFF) [file pcbi.1010358.s015.tiff]

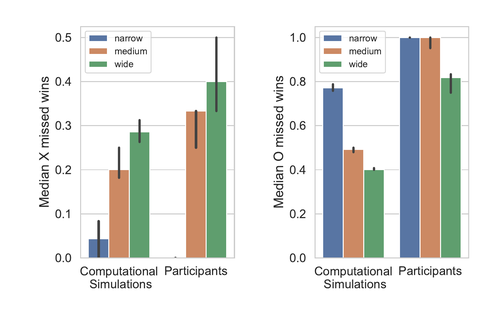

Supplement: S15 Fig — As the shutter size increases, the likelihood of missing winning ‘O’ moves reduces, while the likelihood of missing winning ‘X’ moves increases. (TIFF) [file pcbi.1010358.s016.tiff]

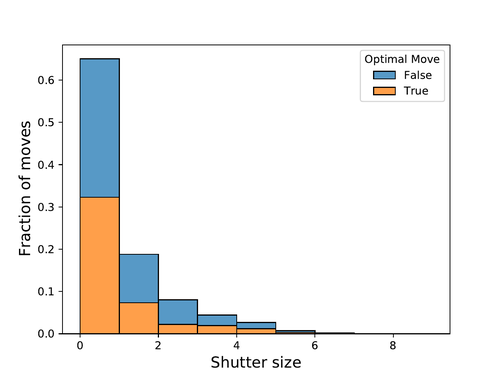

Supplement: S16 Fig — (TIFF) [file pcbi.1010358.s017.tiff]

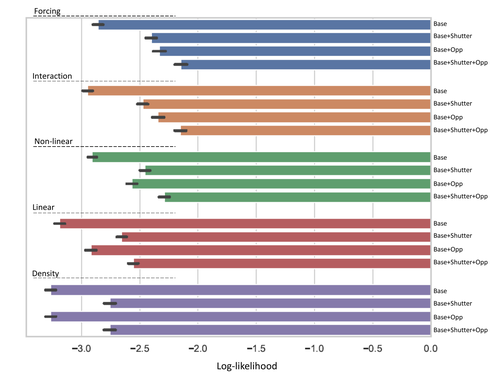

Supplement: S17 Fig — There are three configurations for each scoring strategy: using the scoring strategy as is (“base”), adding a shutter (based on best fit to participants), and further augmenting the scoring strategy with both a shutter and decreased attention to the opponent (based on best fit to participants). For all scoring strategies, adding the shutter significantly improved log-likelihood (in all cases p < 10−5 using a likelihood ratio test which accounts for the additional parameter), and except for the Density strategy (where opponent weight does not have a meaning), augmenting the scoring strategy with decreased attention to the opponent further increased the log-likelihood (2p < 10−5 using a likelihood ratio test which accounts for the additional parameter). (TIFF) [file pcbi.1010358.s018.tiff]

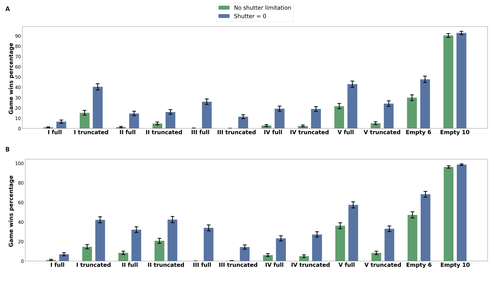

Supplement: S18 Fig — A) Game win percentage of the deep learning models against MCTS with 1000 simulations; B) Game win percentage of the deep learning models against MCTS with 500 simulations. All differences were statistically significant with p < 10−5. (TIFF) [file pcbi.1010358.s019.tiff]
